# Field-Realistic Tylosin Exposure Impacts Honey Bee Microbiota and Pathogen Susceptibility, Which Is Ameliorated by Native Gut Probiotics

**DOI:** 10.1128/spectrum.00103-21

**Published:** 2021-06-23

**Authors:** J. Elijah Powell, Zac Carver, Sean P. Leonard, Nancy A. Moran

**Affiliations:** a Department of Integrative Biology, University of Texas, Austin, Texas, USA; Broad Institute

**Keywords:** dysbiosis, honey bee, microbiota, probiotics, tylosin

## Abstract

Antibiotics have been applied to honey bee (Apis mellifera) hives for decades to treat Paenibacillus larvae, which causes American foulbrood disease and kills honey bee larvae. One of the few antibiotics approved in apiculture is tylosin tartrate. This study examined how a realistic hive treatment regimen of tylosin affected the gut microbiota of bees and susceptibility to a bacterial pathogen. Tylosin treatment reduced bacterial species richness and phylogenetic diversity and reduced the absolute abundances and strain diversity of the beneficial core gut bacteria Snodgrassella alvi and *Bifidobacterium* spp. Bees from hives treated with tylosin died more quickly after being fed a bacterial pathogen (Serratia marcescens) in the laboratory. We then tested whether a probiotic cocktail of core bee gut species could bolster pathogen resistance. Probiotic exposure increased survival of bees from both control and tylosin-treated hives. Finally, we measured tylosin tolerance of core bee gut bacteria by plating cultured isolates on media with different tylosin concentrations. We observed highly variable responses, including large differences among strains of both *S. alvi* and *Gilliamella* spp. Thus, probiotic treatments using cultured bee gut bacteria may ameliorate harmful perturbations of the gut microbiota caused by antibiotics or other factors.

**IMPORTANCE** The antibiotic tylosin tartrate is used to treat honey bee hives to control Paenibacillus larvae, the bacterium that causes American foulbrood. We found that bees from tylosin-treated hives had gut microbiomes with depleted overall diversity as well as reduced absolute abundances and strain diversity of the beneficial bee gut bacteria Snodgrassella alvi and *Bifidobacterium* spp. Furthermore, bees from treated hives suffered higher mortality when challenged with an opportunistic pathogen. Bees receiving a probiotic treatment, consisting of a cocktail of cultured isolates of native bee gut bacteria, had increased survival following pathogen challenge. Thus, probiotic treatment with native gut bacteria may ameliorate negative effects of antibiotic exposure.

## INTRODUCTION

Colonies of the Western honey bee (Apis mellifera) are an actively cultivated agricultural resource accounting for ∼$11.7 billion (2009) in pollination services in the United States alone (1). The simple bee gut microbiome (BGM) provides multiple beneficial effects for honey bees and their colonies (2), ranging from endocrine stimulation that affects feeding and weight gain (3) to immune stimulation and enhanced resistance to infection by pathogens (4–6). The BGM resides in the two regions of the adult worker hindgut: the ileum (a narrow furrowed tube) and the rectum. The ileum is dominated by host-restricted Gram-negative bacteria, including Gilliamella apicola, Gilliamella apis, Snodgrassella alvi, Frischella perrara, and Bartonella apis. The rectum is dominated by host-restricted Gram-positive bacteria, including two bee-restricted *Lactobacillus*-related clades (*Bombilactobacillus* spp. and *Lactobacillus* nr. *melliventris*, previously referred to as Firm-4 and Firm-5, respectively) (7) and *Bifidobacterium* species (B. asteroides, B. indicum, and B. coryneforme) (*Actinobacteria*). This core community is markedly consistent in honey bees worldwide (8–13), and comparisons with microbiomes of related bee species indicate that it has evolved with hosts for millions of years (14).

In agricultural contexts, honey bees are exposed to a variety of chemicals either indirectly, such as exposure to herbicides during foraging, or directly, such as exposure to antibiotics and fungicides used within bee hives in apiculture. How these chemicals impact the BGM is an active area of research. Exposing honey bee workers to agrochemicals, such as the herbicide glyphosate or the antibiotic oxytetracycline (OTC), at high levels in a laboratory setting or at standard treatment levels in hives disrupts microbial community composition, can cause increased susceptibility to an opportunistic pathogen, and can reduce worker survivorship in hives (15–19). Similarly, penicillin-streptomycin treatment increases susceptibility to a microsporidian parasite (20).

Antibiotics, including OTC, have been used for decades in the United States as prophylactic treatments for the highly infectious disease American foulbrood (21), caused by the Gram-positive bacterium Paenibacillus larvae (22–24). Infected bee larvae die and become reservoirs of spores that spread infection throughout the hive and apiary. Spores maintain viability for years (25), and afflicted colonies are typically destroyed via incineration or other means (26, 27).

The overuse of OTC has led to widespread tetracycline resistance (28, 29), including the emergence of P. larvae strains (30, 31) harboring resistance loci that are nearly identical to those of gut microbiome members, suggesting recent horizontal gene transfer (30–32). To circumvent OTC resistance, other antibiotics have been explored for control of American foulbrood (33). Tylosin tartrate, a bacteriostatic macrolide antibiotic used in many veterinary applications (34–36), was approved for use in beehives by the US Food and Drug Administration in 2005 (21CFR520.2640). Tylosin tartrate is effective against OTC-resistant P. larvae and is not acutely toxic to adult or larval bees (37–39). Tylosin does not kill P. larvae spores and can persist in bee products such as honey, which presents issues for human health and product quality (40). While OTC has been shown to disrupt the beneficial gut microbiota and negatively affect bee health (17), we do not know the effects of tylosin on the bee gut microbiome.

Bacterial probiotics have recently been explored as alternative treatments for bacterial and microsporidian infections in honey bee colonies (41–43). Studies of probiotic supplements for P. larvae infections have focused on cultured mixtures of *Lactobacilli* (41, 44). Most studies have used these mixtures as prophylactics, but Daisley et al. (45) used a probiotic mixture after OTC treatments of P. larvae-infested hives and suggested that treated hives eliminated P. larvae faster. A consensus on the efficacy of probiotic treatments for bees is yet to develop, as some studies find little benefit (44) while others claim major benefits (18, 45). To date, studies have used *Lactobacilli* isolates originating from the honey bee foregut (46) or from various environmental sources, including some commonly used in human probiotic formulations. The use of isolates from the native honey bee hindgut community, either as a prophylactic or postantibiotic supplement, has not been explored, though there are several studies showing the stimulation of antimicrobial peptides and immune pathways in the host by members of the BGM (4–6).

In this study, we found that the recommended hive treatment regimen of tylosin tartrate altered the species and strain diversity of gut communities and decreased the absolute abundance and strain diversity of beneficial BGM species. These recommended treatments made bees more susceptible to the opportunistic bacterial pathogen Serratia marcescens (47). Oral inoculation with a probiotic, consisting of a mixture of cocultured BGM members, moderately improved survivorship following pathogen challenge for workers from both control and treated hives. This probiotic community establishes and persists in the gut and suppresses growth of S. marcescens in age-controlled worker bees, as did some combinations of isolates from the BGM.

## RESULTS

### Standard tylosin treatment of hives disrupts gut microbial communities.

**FIG 1:**
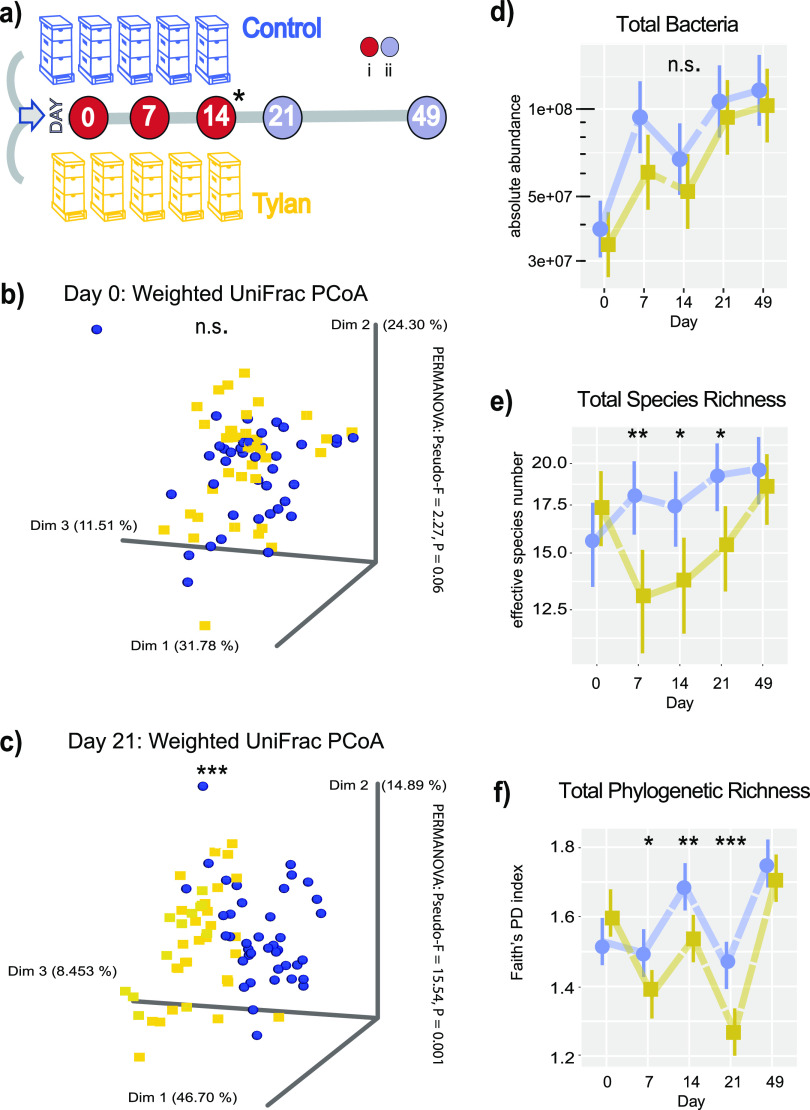
16S rRNA gene metabarcoding analysis of the effect of tylosin tartrate treatment of hives on the microbiome of honey bee workers. (a) Experimental design scheme: 5 hives were given either powdered sugar (control) or tylosin with sugar. All circles indicate days that bees were sampled: (i) red circles are days treatments were also administered, (ii) gray circles indicate days sampled posttreatment, * indicates that only 16S rRNA gene metabarcoding was analyzed for day 14 and not taxon-specific gene targets. (b and c) Principal-coordinate analysis of weighted UniFrac dissimilarity of control and tylosin samples at day 0 and day 3 sampling. Significant clustering by group was analyzed by PERMANOVA. (d) Plot of absolute abundance of bacteria. Total 16S rRNA gene copies were estimated by qPCR and corrected for rRNA operon number per genome. (e) Plot of species richness as assessed by effective species number. (f) Total phylogenetic richness as measured by Faith’s phylogenetic diversity (PD) index. (d to f) Bars on plots represent 95% confidence intervals. Generalized linear mixed-effects models assuming Poisson regression (d) and default distribution (e to f) were used to compare changes in bacterial abundances between control and treatment bees per sampling time. Mixed models were fitted using the package lme4 and followed by *post hoc* tests using package emmeans. *, *P*  ≤  0.05; **, *P*  ≤  0.01; and ***, *P*  ≤  0.001. Blue, control; yellow, tylosin treatment.

**FIG 2:**
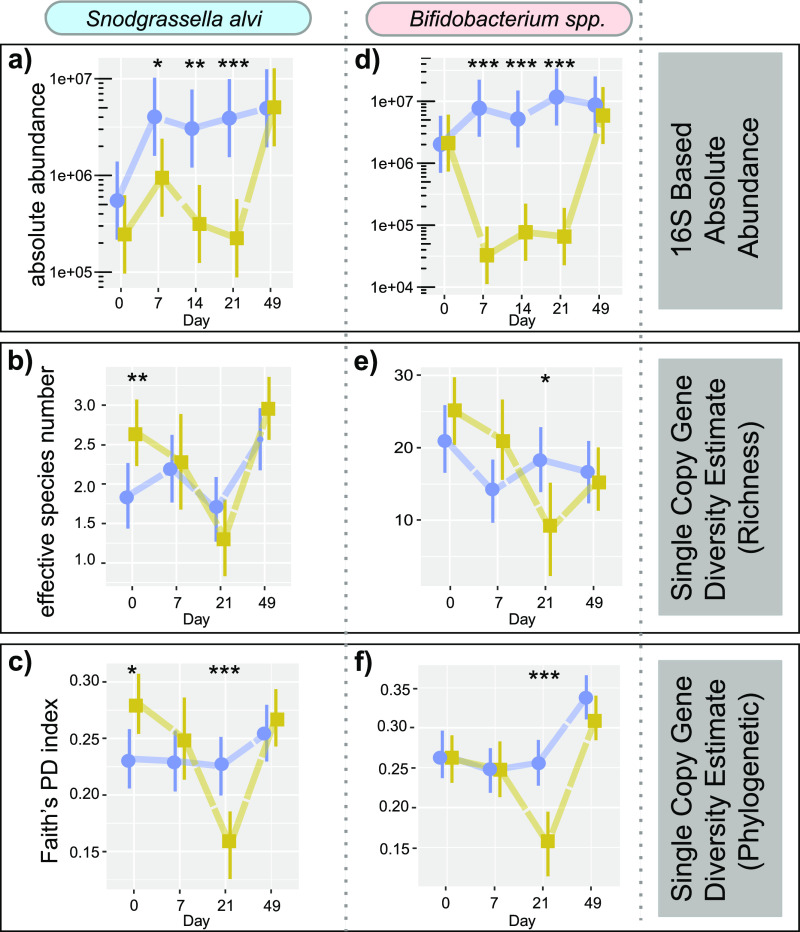
Estimates of 16S rRNA gene-based absolute abundance and single-copy gene target-based alpha diversity (effective species number and phylogenetic diversity) for Snodgrassella alvi (a to c) and *Bifidobacterium* spp. (d to f). Bars on plots represent 95% confidence intervals. Generalized linear mixed-effects models assuming Poisson regression (a and d) and default distribution (b, c, e, and f) were used to compare changes in bacterial abundances between control and treatment bees per sampling time. Mixed models were fitted using the package lme4 and followed by *post hoc* tests using package emmeans. *, *P*  ≤  0.05; **, *P*  ≤  0.01; and ***, *P*  ≤  0.001. Blue, control; yellow, tylosin treatment.

The gut microbial communities of bees sampled from hives that were treated with the commercial formulation of tylosin tartrate differed from those of control hives, as assessed by beta diversity analysis (weighted UniFrac principal-coordinate analysis [PCoA] and permutational multivariate analysis of variance [PERMANOVA]) of 16S rRNA gene metabarcode data (Fig. 1a to c; Table 1). Beta diversity comparison between control and treated hives over the course of antibiotic treatment revealed increasing divergence of treatment groups as the treatment progressed (Table 1), with the greatest effect at day 21, 1 week after the final antibiotic treatment. Community analysis techniques (ANCOM) indicated that suppression of the proportions of *S. alvi* and *Bifidobacterium* amplicon sequence variants (ASVs) in tylosin-treated hives drove the observed differences in beta diversity. Differences in the absolute abundances of these species are detailed below (Fig. 2) and in Fig. S1 in the supplemental material.

**TABLE 1:** Pairwise PERMANOVA results of weighted UniFrac distances of samples in treatment groups by sampling day^*a*^

Day	No. of samples	Pseudo-*F* value	*P* value	*q* value
0	80	2.27	0.055	0.055
7	77	2.64	0.046	0.046^*b*^
14	80	7.93	0.001	0.001^*c*^
21	80	15.54	0.001	0.001^*c*^
49	79	0.62	0.631	0.631

a Groups compared were control versus tylosin treated, with 999 permutations.

b *q*  ≤  0.05.

c *q*  ≤  0.001.

The absolute abundance of total bacteria per bee did not differ significantly between control and treated hives at any time during the experiment (Fig. 1d). In spite of similar community sizes, alpha diversity richness, as reflected by effective species number (ESN) and by phylogenetic diversity (PD), was significantly lower in treated hives starting from the first posttreatment sampling (day 7) through day 21 (1 week post-final treatment). Diversity in treated hives rebounded between this first posttreatment sampling and 28 days later (day 49), when gut communities in control and treated hives had similar alpha and beta diversity levels.

Other shifts in the microbiota composition of bees in the apiary occurred over the course of the experiment, potentially caused by seasonal changes in floral food sources (48, 49). These trends included an overall upward trend in the absolute size and diversity of the microbial community (Fig. 1d to f) driven by increases in the absolute abundance of *Bombilactobacillus* and *Lactobacillus* nr. *melliventris* (see Fig. S2) as well as drops in abundances in the *Commensalibacter* group (also known as Alpha -2.1) (see Fig. S3a). Additionally, *B. apis* exhibited an expansion from days 7 to 21 but was reduced again by the 49-day time point (Fig. 1a and b; Fig. S3).

### Both absolute numbers and strain diversity of *S. alvi* and *Bifidobacterium* were suppressed by tylosin treatment.

Comparison of absolute abundance of *S. alvi* and *Bifidobacterium* spp., based on metabarcoding and quantitative PCR (qPCR) of 16S rRNA gene sequences, revealed significant decreases in both groups in the treated hives (Fig. 2a and d). These differences were apparent at day 7, continued throughout the course of treatment, and reached their most extreme by day 21.

Metabarcoding of taxon-specific single-copy genes was used to examine the changes in strain diversity within these groups as well as within *Gilliamella* spp. Interestingly, species-level suppression of *S. alvi* and *Bifidobacterium* spp. was apparent in the asymmetrical amplification of these targeted genes (Table 2), with many samples failing amplification for these taxa in the treated hives and far less amplification in the control hives. ESN was very low in *S. alvi*, ranging from 1.68 to 2.57 in the control group, and differences between *S. alvi* ESNs in treated and control groups were slight, with the greatest difference on day 0, prior to the start of treatment (Fig. 2b). In contrast, ESN of *Bifidobacterium* spp. ranged from 14.01 to 21.23 and was significantly diminished in treated hives at day 21 (Fig. 2). Both *S. alvi* and *Bifidobacterium* had significantly depressed phylogenetic diversity in treated hives at day 21 (Fig. 2c and f), the time point at which maximum differences in whole community composition were observed based on 16S rRNA gene profiles. *Gilliamella* spp. as a group were also assessed at the strain level and did not experience suppression in absolute numbers or in alpha diversity metrics (see Fig. S4). Also of note, *F. perrara* was diminished (Fig. S3a) in the tylosin-treated hives, and this suppression continued to the last sampling point (day 49). *Commensalibacter* spp. followed a similar trend in both treatment groups but started out significantly higher in the tylosin-treated group prior to treatment and until the day 7 sampling and was significantly lower thereafter (Fig. S3c).

**TABLE 2:** Number of samples included for each gene target by treatment group^*a*^

Day	No. of samples							
	16S rRNA gene		*S. alvi* (*minD*)		*Gilliamella* spp. (*rimM*)		*Bifidobacterium* spp. (*groEL*)	
	CTL	TYL	CTL	TYL	CTL	TYL	CTL	TYL
0	40	40	34	33	27	34	31	31
7	39	38	32	16^*b*^	29	31	37	16^*b*^
14	40	40						
21	40	40	35	25	40	38	33	16^*b*^
49	39	40	38	37	40	40	17	23

a Samples that did not amplify or have sufficient reads pass quality control for analysis were not included. Group proportions were compared to 16S amplifications by one-way Fisher’s exact test. CTL, control; TYL, tylosin treated.

b *P* < 0.05.

We analyzed taxon-specific single-copy genes for beta diversity using weighted UniFrac, which generates a multivariate dissimilarity matrix based on reads for each ASV and takes into account phylogenetic relatedness. We did not observe clustering by group during the treatment course. These observations taken together (absolute abundance and alpha and beta diversity of specific taxa) indicate that tylosin treatment reduces the absolute numbers of *S. alvi* and *Bifidobacterium* spp. along with the number of strains and their phylogenetic diversity. However, the lack of clustering in weighted UniFrac principal-coordinate analyses indicate that these altered communities did not take on consistent compositions.

### Workers from tylosin-exposed hives were more likely to die following challenge by a bacterial pathogen.

**FIG 3:**
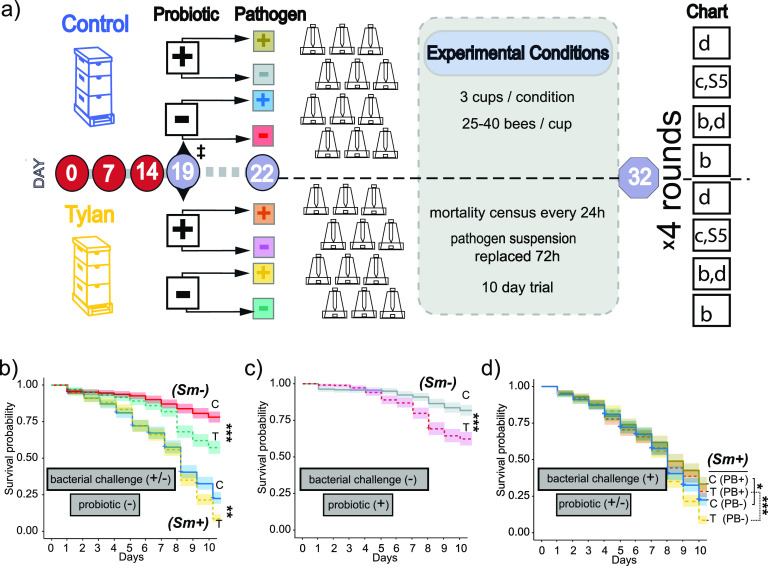
Bacterial pathogen challenge following hive tylosin treatment and/or probiotic treatment. (a) Matched hives were treated with sugar (control) or sugar with antibiotic (tylosin) on days 0, 7, and 14. One hive per condition (control or tylosin) per round (4 rounds total) was used. Workers were removed to the lab on day 19 (‡) and split into two groups per hive/treatment condition. One of these groups was fed a probiotic mixture of cultured bee gut specific microbiota (PB+) and the other was fed buffer only (PB−). Bees were allowed 3 days before all groups were split again, and within each split, one half were assigned to receive a pathogenic challenge (*Sm*+) in sugar syrup while the other split received only sugar syrup (*Sm*−). All split groups were placed in cup cages of 25 to 40 individuals (3 cups per condition). Mortality was assessed over the next 10 days with the pathogen suspension being replaced every 72 h. (b) Kaplan-Meier curve of survival probability of combined control and tylosin trials that were not provided probiotic (PB−) with and without pathogenic challenge (*Sm*+/*Sm*−). Control groups had significantly higher survival probabilities than tylosin-treated groups. Unchallenged (*Sm*−) groups survived better than challenged groups (Sm+). (c) Unchallenged (*Sm*−) control bees provided probiotic (PB+) survived better than unchallenged tylosin-treated bees given probiotic (PB+), though these results are similar to those for bees not given probiotic (see Fig. S5 in the supplemental material). (d) Following pathogen challenge (*Sm*+), probiotics improved survival probabilities; that is, control PB+ survived better than control PB−, and tylosin-treated PB+ did much better than tylosin-treated PB− (for each combined survival data plot, Cox proportional hazards mixed effects model [by trial]). *, *P* < 0.05; **, *P* < 0.01; ***, *P* < 0.001.

Adult worker bees were sampled from paired treated and untreated hives at the Driftwood site 5 days after the standard 3-week tylosin treatment and taken back to the lab. After 3 days in the lab, cohorts of each group were fed either a sugar suspension alone or one containing an opportunistic Gram-negative pathogen (Serratia marcescens strain N10A28). They were then split into cages and observed for 10 days (Fig. 3a). This scheme was duplicated with bees that had been fed a probiotic mixture of cultured BGM members during the 3 days prior to bacterial challenge. This experiment was conducted 4 times using different paired hives for each trial.

In the cohort that did not receive the probiotic supplement (Fig. 3b), untreated control bees had a statistically significant higher probability of survival than tylosin-treated bees whether challenged (*Sm*+) or not (*Sm*−) with a bacterial pathogen. As expected, pathogen treatment increased mortality, with >75% of both groups dying by the end of the study compared to <60% in the nonchallenged groups.

### A probiotic treatment of cultured BGM isolates increased survival in both control and tylosin-treated bees following pathogen challenge and had no effect on survival for nonchallenged bees.

The results from bees fed the probiotic mixture (PB+) and not challenged with a pathogen (*Sm*−) mirrored the unchallenged control and tylosin-treated bees from the nonsupplemented group (Fig. 3b and c; Fig. S5). Thus, in the absence of the pathogen, the survival probabilities were similar for control and tylosin-treated bees, whether or not they received the probiotic, and were similar between the two supplementation scenarios, with the control group surviving slightly better than the antibiotic-treated group.

When challenged with a bacterial pathogen (*Sm*+ in Fig. 3d), bees receiving the probiotic had significantly higher survival probabilities whether or not they had been exposed to tylosin. These improved survival probabilities are reflected in total survival among all 4 trials at 10 days. For bees not exposed to tylosin and receiving the probiotic, survival was 33% (145 survivors of 433 subjects) versus 19% (89 survivors of 480 subjects) for those not receiving the probiotic. For tylosin-exposed bees, 10-day survival was 28% (123 survivors of 433 subjects) for those receiving the probiotic versus 9% for those not receiving the probiotic (41 survivors of 480 subjects). Thus, for antibiotic-exposed bees, the probiotic treatment resulted in a 3-fold improvement in survival.

### Different taxonomic lineages and strains within lineages respond differently to tylosin.

**FIG 4:**
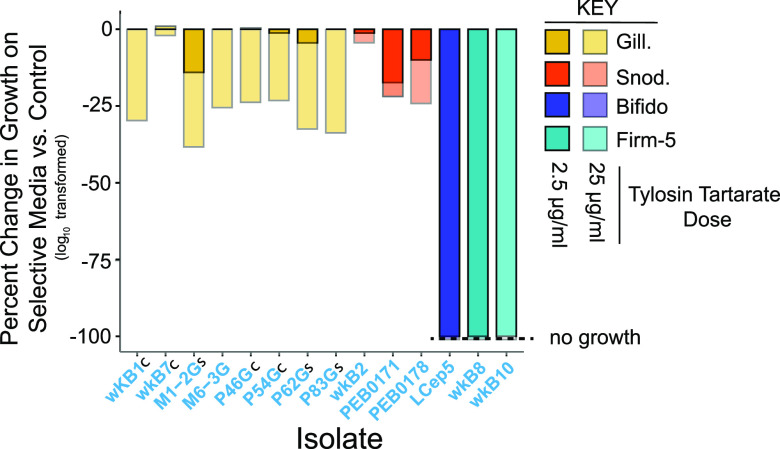
Impact of tylosin tartrate on growth of bee gut microbiota isolates. Suspensions of bacterial isolates were serially diluted and spotted onto triplicate plates of either CBA alone or CBA with tylosin tartrate (2.5 or 25 μg/ml). After incubation, colonies were counted and viable cell counts calculated. Viable CFU counts were then log_10_ transformed, and the percent change in viability from the control to selective platings was calculated. *Bifidobacterium* and Firm-5 (*Lactobacillus* nr. *melliventris*) did not grow at either tylosin tartrate concentration. *Gilliamella* isolates marked with a letter have been identified to the species level (c, *G. apicola*; s, *G. apis*).

We plated 10-fold spot dilutions of cultured isolates onto culture plates with two concentrations of tylosin tartrate (2.5 μg/ml and 25 μg/ml) and with no tylosin tartrate and then compared log_10_ transformed counts of bacterial growth on plates with different levels of antibiotic (Fig. 4). Gram-positive cultures (*B. asteroides* and *Lactobacillus* nr. *melliventris*) were completely suppressed with no growth occurring at any antibiotic concentration. In contrast, *G. apis*, *G. apicola*, and *S. alvi* strains had levels of suppression that increased with increasing tylosin tartrate concentration. However, a single strain of *G. apicola* (strain wkB7) and of *S. alvi* (strain wkB2) experienced only minor suppression (<5%) even with the high dose.

### The probiotic defined community is able to stably reside in the bee gut.

**FIG 5:**
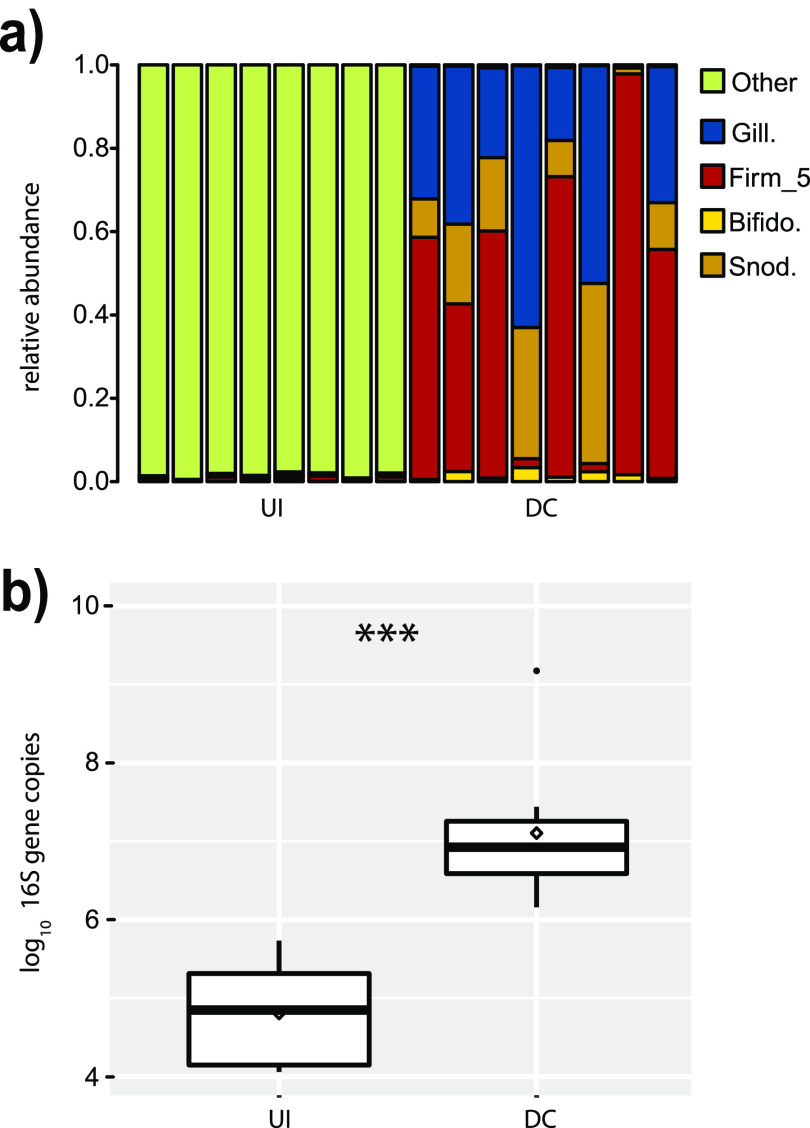
Stability of defined community at 5 days postinoculation. Bees were allowed to emerge on a frame and either placed directly in a cup cage (uninoculated [UI]) or first fed 5 μl of a defined community inoculum before caging (defined community [DC]) at an of OD_600_ of 1. Bees were fed sterile sucrose and gamma irradiated pollen until day 5, when DNA was prepared from dissected guts. (a) Bar plot of uninoculated workers and those fed cocultured defined community as assessed by 16S V4 metabarcoding. Uninoculated bees were overwhelmingly (>90%) infected with Staphylococcus. (b) Log_10_ 16S gene copies as assessed by qPCR. ***, *P* < 0.0001, Wilcoxon.

We compared the microbial populations of bees who were uninoculated to those of bees who were fed the probiotic consisting of the defined community. We assessed both composition and size and found that bees retained large populations of the BGM members in the defined community at 5 days postinoculation (Fig. 5).

### The probiotic defined community and some combinations of its members are able to suppress replication of a bacterial pathogen within the guts of adult honey bees.

**FIG 6:**
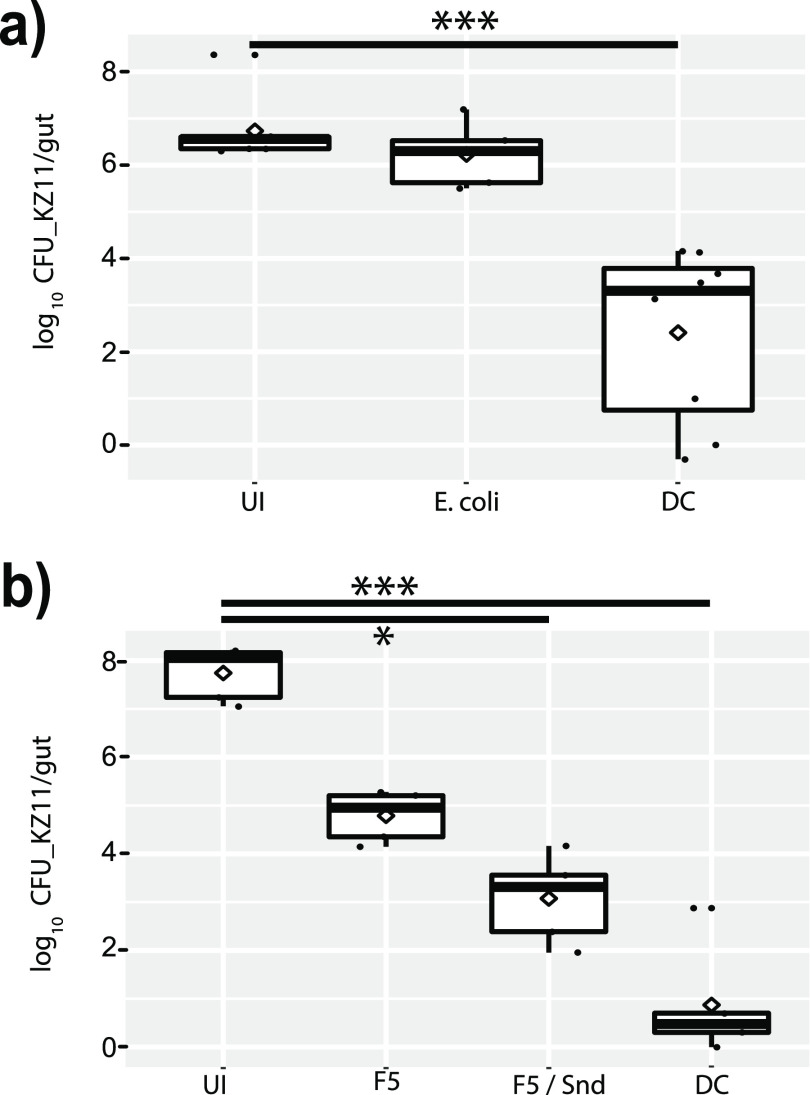
CFU of the bacterial pathogen Serratia marcescens KZ11 (kanamycin resistant) per plated bee gut. (a) Recovered colonies of S. marcescens strain KZ11 from either uninoculated bees (UI), those infected with E. coli, or the full defined community (DC). (b) Recovered colonies of S. marcescens KZ11 from either uninoculated bees (UI), those infected with *Lactobacillus* nr. *melliventris* wkB8 and 10 (F5), F5 and *S. alvi* (F5/Snd), or the full defined community (DC). *, *P* < 0.05; ***, *P* < 0.001, *post hoc* pairwise comparisons using Tukey and Kramer (Nemenyi) test with Tukey distribution approximation for independent samples following Kruskal-Wallis.

We inoculated newly emerged worker bees with combinations of the defined BGM, Escherichia coli, or nothing in the case of the control. We later exposed these bees to S. marcescens KZ11, modified to carry a kanamycin resistance marker. A day later, we recovered the guts from these bees, plated serial dilutions, and found that the full defined probiotic community greatly reduced the number of viable colonies of the pathogen relative to those in both control bees and bees fed a nonspecific bacterial species (E. coli). Bees that had been inoculated with the combination of *Lactobacillus* nr. *melliventris* strains and *S. alvi* also suppressed the pathogen (Fig. 6).

## DISCUSSION

Antibiotics have been a useful tool in beekeeping for controlling P. larvae, but extensive prophylactic use leads to resistance and increases the frequency of resistance genes in environmental bacteria. Recently, several lines of evidence have pointed to a role of the gut microbiota in the health of honey bees (3), raising the possibility that antibiotic treatments can harm hives by disrupting the gut microbiota of workers. While previous studies of apiculture antibiotics have exposed bees to high concentrations of purified OTC in laboratory settings (17), our study examined whole-hive treatments with a commercial preparation of tylosin tartrate at the recommended application levels and treatment regime.

Our experiments yielded three major findings. First, tylosin treatments had a major sustained impact on community composition, including lowered absolute and relative abundances of two core species clusters: *S. alvi* and *Bifidobacterium* spp. This finding is not entirely unexpected but is the first demonstration that typical, recommended hive-level treatments are disruptive to beneficial gut bacteria of honey bees. The effects were evident at both the species and strain levels. The impact of tylosin treatment persisted during the treatment period but diminished afterwards, with treated and control hives showing no significant differences a month later. Some of the treated hives failed to yield any PCR amplicons with the species-specific primers, suggesting that certain species were essentially eliminated from bees in treated hives. The treated bees also showed lower survival probabilities when brought into the laboratory, even without a pathogen challenge, suggesting that microbiota disruption due to tylosin exposure lowers general vigor. Previous studies have shown that the bee gut microbiota has multiple roles in host health, with effects on development, hormonal signaling, nutrition, and immunity (3–6, 50–54). The impact of microbiota disruption on bee survivorship probabilities could reflect any combination of these roles.

Second, we found that probiotic treatment with a cocultured mixture of native bee gut strains boosted survivorship of bees from tylosin-treated and control hives following challenge by an opportunistic bacterial pathogen, S. marcescens. This effect was moderate for control hives and greater for tylosin-treated hives. Previous studies have shown some benefits from probiotic treatments of environmental strains of *Lactobacillus* and other bacterial species (18, 45), but those studies did not incorporate species from the dominant bee gut taxa. In some cases, probiotic supplements intended to suppress pathogens have proved somewhat pathogenic themselves, decreasing bee survivorship (55). Our experiments used a cocultured defined community containing only strains representing the typical dominant taxa in adult worker hindguts. These bacteria are naturally transmitted among workers within hives, colonizing soon after adult emergence and persisting through the host life span (56). Thus, we expected their use as a probiotic treatment would have a persistent beneficial impact on gut communities within hives. Importantly, the probiotic treatment in itself did not negatively affect bee survivorship in the absence of pathogen challenge, as assessed for bees from natural conditions in hives. These benefits were evident even in a randomly sampled population of bees of mixed ages. Bees of differing ages engage in hive tasks expected to result in differential exposure to hive treatments, and young bees, in the process of acquiring a microbiota, could be impacted more than older bees with an established microbiota.

A third finding of our study is that species and strains within the bee gut microbiota show dramatically different sensitivities to tylosin, as assayed in laboratory cultures. Most striking, strains of the core species, *G. apicola*, and *S. alvi*, differed strongly in sensitivity to the compound. This observed variation provides a likely explanation for why strain diversity declines in treated hives, as some strains are more able to persist while others decline following treatments. Furthermore, the observed variation may inform the design of probiotics for bees. An earlier study showed that glyphosate can impact the gut communities of bees, with *S. alvi* particularly susceptible; however, one strain, *S. alvi* wkB2, was much less impacted (15, 16). Interestingly, *S. alvi* wkB2 was also the *S. alvi* strain we found to be insensitive to tylosin. Thus, selecting naturally occurring gut symbiont strains that are resistant to antibiotics or other chemicals may improve the effectiveness of probiotic treatments.

Tylosin is a macrolide antibiotic that affects almost all Gram-positive and some Gram-negative species (57). Indeed, we observed that it completely repressed all tested strains of the Gram-positive species in the bee gut microbiota, including *Bombilactobacillus* spp., *Lactobacillus* nr. *melliventris*, and *Bifidobacterium* spp. Based on these culturing data, tylosin treatment would be expected to have a large impact on the abundance of *Lactobacillus*-related strains *in vivo*. Surprisingly, however, the absolute abundances of the *Bombilactobacillus* spp. and *Lactobacillus* nr. *melliventris* clades were not significantly impacted by tylosin treatment of hives. Potentially, our tested laboratory isolates did not include resistant strains that exist in natural bee gut communities. Alternatively, the localization of *Lactobacillus*-related strains in the distal hindgut (rectum) may somehow reduce exposure of these symbionts to the antibiotic.

A fourth finding is that this defined community is stable in bees 5 days after inoculation. Thus, this introduced mix of cultured native bee microbiota is not transitory and is able to persist in the gut over time. Potentially, it augments displaced lineages in the microbiome.

Finally, we have demonstrated that inoculation by different members and combinations of members of the defined community can lessen the magnitude of pathogenic bacterial infections. The most protective combination is the full defined community, which almost completely suppressed S. marcescens within bees. The combination of *S. alvi* with *Lactobacillus* nr. *melliventris* was also highly protective. We did not investigate the mechanisms of this suppression, which could involve direct microbial interactions, stimulation of the host immune system, or some combination of these mechanisms.

Models of bee colony dynamics in the face of environmental stressors or infectious agents show that changes in worker survivorship can determine whether a colony collapses or survives (58). In particular, for infectious agents, the rate of transmission within the colony has a major impact (59). High pathogen loads in workers lacking a robust BGM, as observed for S. marcescens, potentially lead to elevated rates of transmission and thereby impact colony fate.

### Conclusion.

Our results suggest that typical use of tylosin tartrate in apiculture may reduce vigor of workers and increase their susceptibility to opportunistic pathogens; potentially, this is a factor in the decline of colonies. This effect likely results from gut dysbiosis, as we observed large effects on gut community composition, including decline in the abundance and strain diversity of beneficial bacterial species. We also show that a probiotic mixture of cocultured isolates of native hindgut species enhanced resistance to a pathogen while having no negative effect on its own. Potentially, such treatments could be developed as a tool for improving colony health. Native gut species vary in resistance to tylosin and glyphosate, and probiotic mixtures might be designed to specifically include strains that are robust in the face of exposure to these or other chemical challenges.

## MATERIALS AND METHODS

### Tylosin tartrate treatment of hives.

For field experiments, we established single deep-box Langstroth-style beehives in an uncultivated pasture in Driftwood, Texas (latitude, 30.1114998; longitude, −98.0212251) in the summer of 2018. We provided control hives (*n* = 5) 20 g of confectioners’ sugar every 7 days for 3 weeks and experimental hives (*n* = 5) 20 g of confectioners’ sugar along with 200 mg Tylan (Elanco Animal Health, IN, USA) (the commercial formulation containing tylosin tartrate) on the same schedule (on days 0, 7, and 14). This schedule follows the antibiotic administration regime mandated by the USDA new animal drug application (NADA-013-076) and is used in commercial apicultural practice. We dusted the powdered sugar with or without antibiotics over the top bars of the hive. We collected samples of workers from all 10 hives as a baseline on the first day of treatment (day 0) before administering the antibiotics and sugar and then on days 7 (2nd treatment), 14 (3rd treatment), 21 (1 week posttreatment), and 49 (35 days after the final treatment and 28 days after the previous sampling point). For each sample, we placed >25 adult workers into sterile 50-ml Falcon tubes and flash froze them on site by placing them into an ethanol/dry ice slurry. We stored these samples at −80°C until they were processed. We avoided collecting newly emerged workers (those lacking established microbiota) by taking samples from pollen and nectar storage frames near the edge of the bee mass and not from brood frames. Sample collection information is presented in Table S1 in the supplemental material.

### DNA extraction.

For each hive and time point collected, we prepared DNA from 8 individual whole bee gut samples via the cetyltrimethylammonium bromide (CTAB)/bead beating technique established in reference 56. We prepared samples in a randomized way across conditions, hives, and collection points to avoid batch effects. We examined resultant DNA extractions on a 1% agarose gel to examine the quality, and quantified DNA with a Qubit instrument and the double-stranded DNA (dsDNA) broad-range (BR) kit (Invitrogen, MA, USA). We diluted the extractions 100-fold prior to qPCR quantitation and high-throughput sequencing strategies. We used less-dilute samples in cases where amplification failed in an attempt to amplify difficult templates.

### High-throughput sequencing of bacterial community.

We took two different approaches to assessing the compositions of bacterial communities in the individual samples: 16S rRNA gene V4 metabarcoding and taxon-specific single copy gene metabarcoding. The 16S rRNA gene V4 metabarcoding technique and primers were similar to those in many studies of overall bacterial community composition (60, 61). The taxon-specific single-copy gene method was used to look at strain variation within specific lineages as in previous studies of bee gut microbiomes (12, 13, 19). For both of these approaches, primer sequences and amplification protocols are listed in in Table S2.

The 16S rRNA gene library building method consisted of a two-part scheme to amplify and barcode the target region for sequencing. We used a similar approach, but with single-copy genes, to characterize strains within bee gut microbial taxa: for *S. alvi* we used the *minD* gene (13), for *Gilliamella* spp. the *rimM* gene (19), and for *Bifidobacterium* the *groEL* gene (62). The single-copy gene metabarcoding was not used for the individually inoculated bees used for the stability experiment. Detailed explanations of these techniques are supplied in Text S1 in the supplemental material.

### Processing and analysis of high-throughput reads.

We analyzed amplicon sequence data with Qiime 2, including steps for quality control, read processing, and compositional and diversity analyses; details are in Text S1 in the supplemental material.

### qPCR for 16S rRNA gene copy abundance.

We used the techniques outlined in previous studies (56, 63) for absolute SYBR green qPCR quantitation of total 16S rRNA gene copies with the 27F/355R (Table S2) universal 16S primer set along with a serially diluted plasmid-based standard.

Resultant 16S rRNA gene copy counts were corrected for dilution, and the estimated absolute abundance for each bacterial species was calculated by multiplying the total number of 16S rRNA gene copies obtained by qPCR by the percent relative abundance of each species, adjusting based on genomic 16S rRNA gene copy number, as in reference 17.

### Pathogen challenge.

We investigated the impact of standard hive-level treatment using the tylosin formulation on the mortality of bees exposed in the lab to the opportunistic pathogen S. marcescens strain N10A28, an isolate from a honey bee gut (GenBank accession CP033623). We also examined whether augmentation of the disrupted bee gut microbiota with a probiotic treatment affected survivorship. A schematic of the experimental set up is included in Fig. 3a and summarized as follows. Two similarly sized hives (as assessed with frames of brood and total frames of workers) were given either the 3-week treatment of tylosin and powdered sugar (as described above) or powdered sugar alone. Five days after the final treatment, hundreds of bees were collected from both hives and brought to the laboratory. At this point, the collected bees from each treatment group were split into 2 subgroups, one of which was inoculated with a probiotic mixture of BGM cocultured isolates (see below) and the other with a sugar–phosphate-buffered saline (PBS) blank. The bees were maintained in cup cages in a 35°C incubator at ∼90% relative humidity for 72 h, and then these 4 groups (control hive ± probiotic and tylosin-treated hive ± probiotic) were then split to 6 cups of 24 to 40 bees per cup per group. Of these 6 cups per group, 3 were provided sterile sugar syrup alone, and 3 cups were given a suspension with an optical density at 600 nm (OD_600_) of 0.5 made from a Columbia agar with sheep blood (CBA) plate of S. marcescens strain N10A28 scraped into 1:1 sugar syrup. This suspension was replaced every 72 h. We recorded and removed dead bees every 24 h for 10 days. We conducted these experiments in 4 separate rounds with different paired hives for each round.

The probiotic mixture was prepared by streaking frozen glycerol stocks of *S. alvi* (wkB2), *G. apicola* (wkB1 and wkB7), *B. asteroides* (LCep5), and *Lactobacillus* nr. *melliventris* strains (wkB8 and wkB10) to Columbia agar (Thermo Scientific, MA, USA) with 5% sheep blood (CBA) and allowing growth for 4 days at 35°C and 5% CO_2_. Resulting colonies from each isolate were scraped into 10 ml of 1× sterile PBS to an OD_600_ of ∼1. These suspensions were combined and spread on fresh CBA plates. They were cultured overnight at 35°C and 5% CO_2_ and then scraped into 1× PBS, which was used to inoculate groups of workers by placing 250 μl of the suspension on the pollen which the workers were allowed to consume.

### Tylosin tartrate treatment of cultured bacterial isolates.

We tested for tylosin tartrate resistance using representative isolates of several bee gut-specific clades: *S. alvi* (wkB2), *G. apicola* (wkB1 and wkB7), *B. asteroides* (LCep5), and *Lactobacillus* nr. *melliventris* (wkB8 and wkB10). These isolates were struck to CBA and allowed to grow for 4 days at 35°C and 5% CO_2_. Resulting colonies from each isolate were scraped into 1× sterile PBS to an OD_600_ of ∼1. We made 10-fold serial dilutions of these suspensions and struck 10-μl spots from all of them to triplicate plates of three conditions: CBA alone (control), CBA with 2.5 μg/ml tylosin tartrate, or CBA with 25 μg/ml tylosin tartrate. These plates were cultured for 4 days at 35°C and 5% CO_2_; resultant colonies were counted and viable CFU were estimated.

### Inoculation of individual worker bees with a defined community.

We tested the stability of the defined community probiotic *in vivo* by feeding 5 μl of the bacterial suspension (at OD_600_ of ∼1) to a cohort of workers that had been allowed to emerge on a caged frame overnight. A control cohort was fed 5 μl of 1× PBS. Bees were then maintained in cup cages as described above for 5 days. After this interval, we individually dissected guts, extracted DNA, and assessed the bacterial community via 16S rRNA gene metabarcoding and the total community size by qPCR, as described above.

### *In vivo* test of pathogen and BGM member interaction.

Sterile day-old emerged workers were fed 5 μl of either PBS (UI), single bacterial isolates, or combinations (Escherichia coli K-12 DH5α, *Lactobacillus* nr. *melliventris* wkB8 and wkB10 [F5], *S. alvi* [Snd], and full defined community [DC]) at an OD_600_ of ∼1. Bees were maintained in cup cages for 3 days, when they were fed 5 μl of kanamycin-resistant S. marcescens strain KZ11 (a strain originally isolated in reference 47 and modified via Tn*5* integration [M. I. Steele, E. V. S. Motta, T. Gattu, D. Martinez, and N. A. Moran, submitted for publication]) at an OD_600_ of ∼1. After 24 h, the guts were removed and homogenized in 100 μl PBS. We then performed 10-fold dilutions and spotted them onto CBA plates with kanamycin at 50 μg/ml. We enumerated colonies after overnight incubation at 35°C. The two trials in which the defined community was used are visualized in Fig. 6. Additional trials are found in Fig. S6.

### Data availability.

Files containing read data for 16S rRNA genes and taxon-specific single-copy gene metabarcodes are available through the NCBI SRA BioProject under accession PRJNA699143.
