# Supplementary material for: Field-Realistic Tylosin Exposure Impacts Honey Bee Microbiota and Pathogen Susceptibility, Which Is Ameliorated by Native Gut Probiotics
Source: Microbiol Spectr. 2021 Jun 23;9(1):10.1128/spectrum.00103-21. doi: 10.1128/spectrum.00103-21 (PMC8552731; doi:10.1128/spectrum.00103-21)
Supplement: SUPPLEMENTAL FILE 1 — Fig. S1 to S6, supplemental text, and Tables S1 to S4. Download SPECTRUM00103-21_Supp_1_seq10.pdf, PDF file, 2.1 MB [file spectrum00103-21_supp_1_seq10.pdf]

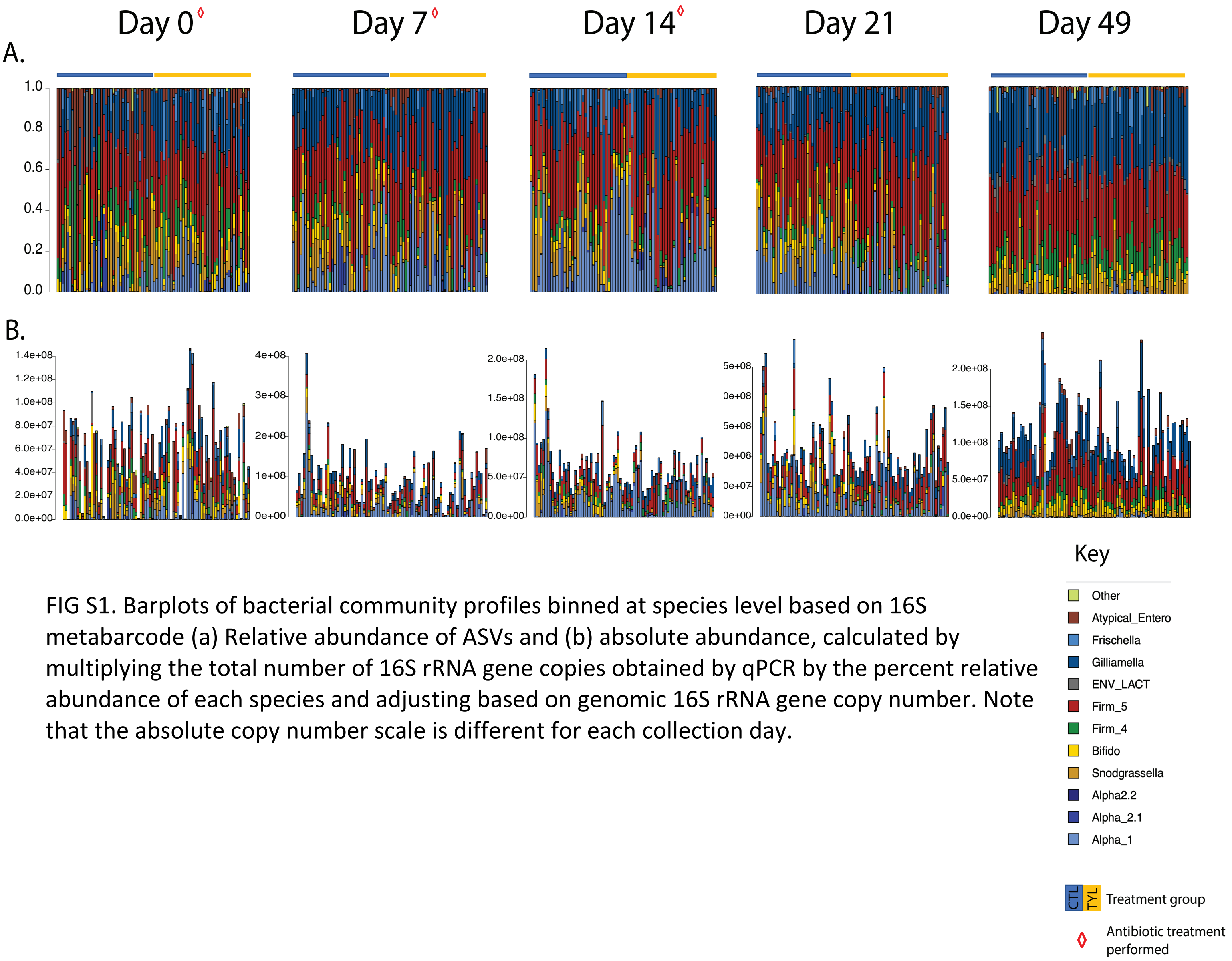

FIG S1. Barplots of bacterial community profiles binned at species level based on 16S metabarcode (a) Relative abundance of ASVs and (b) absolute abundance, calculated by multiplying the total number of 16S rRNA gene copies obtained by qPCR by the percent relative abundance of each species and adjusting based on genomic 16S rRNA gene copy number. Note that the absolute copy number scale is different for each collection day.

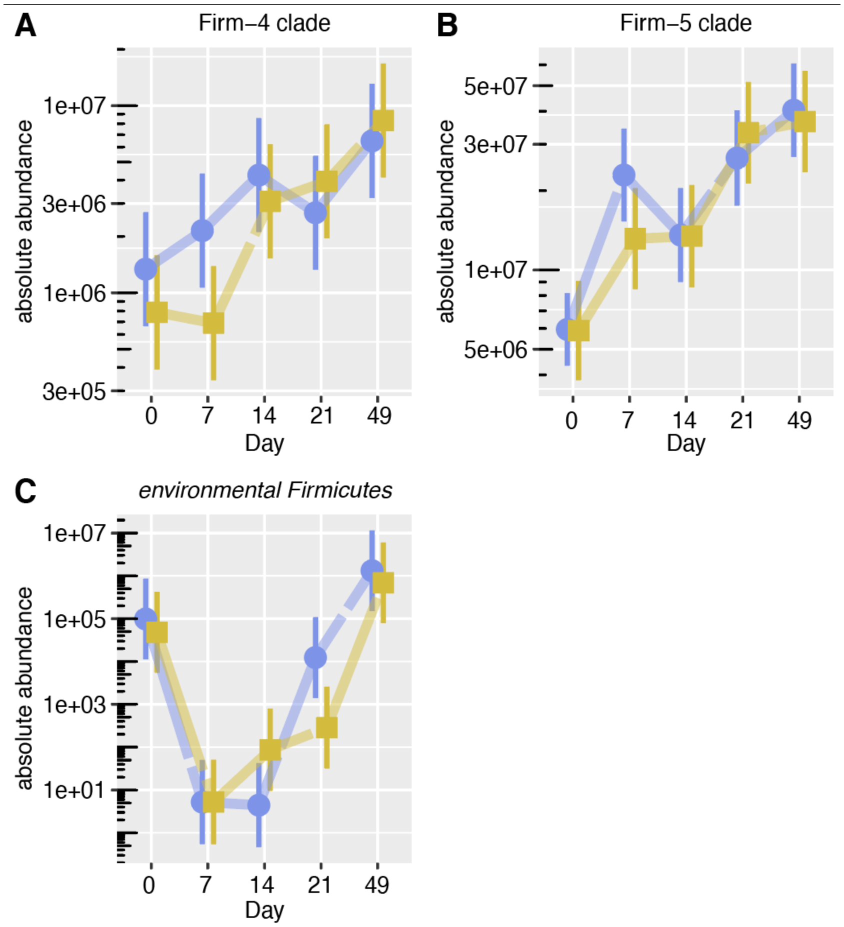

FIG S2. Absolute abundance of Firmicutes groups by sampling day. (A) BGM-associated Firm-4 clade. (B) BGM-associated Firm-5 clade. (C) environmentally associated Firmicutes. Generalized linear mixed-effects models assuming Poisson regression were used to compare changes in absolute bacterial abundances between control and treatment hives per sampling time. Mixed models were fitted using the package *lme4* and followed by post hoc tests using the package *emmeans*. *, P≤0.05; **, P≤0.01; and ***, P≤0.001. Blue=Control; Yellow=Tylosin treatment.

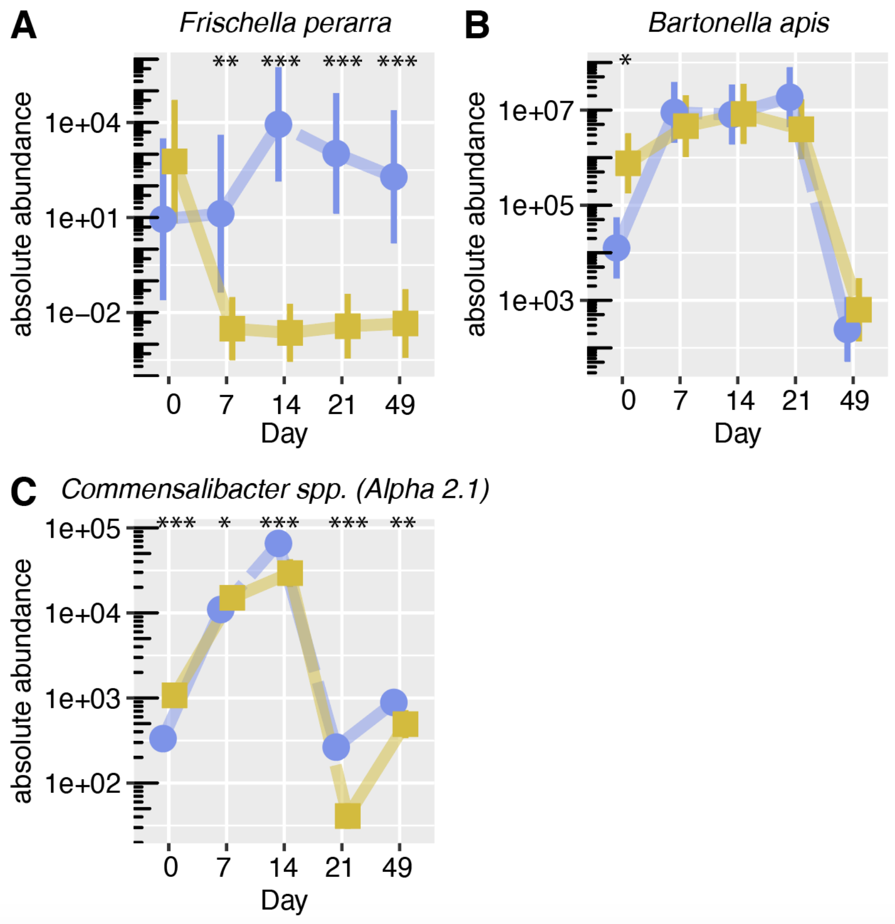

FIG S3. Absolute abundance of additional groups by sampling day. (A) Frischella perrara, (B) Bartonella apis, and (c) Commensalibacter spp. Generalized linear mixed-effects models assuming Poisson regression were used to compare changes in absolute bacterial abundances between control and treatment hives per sampling time. Mixed models were fitted using the package *lme4* and followed by post hoc tests using the package *emmeans*. *, P≤0.05; **, P≤0.01; and ***, P≤0.001. Blue=Control; Yellow=Tylosin treatment.

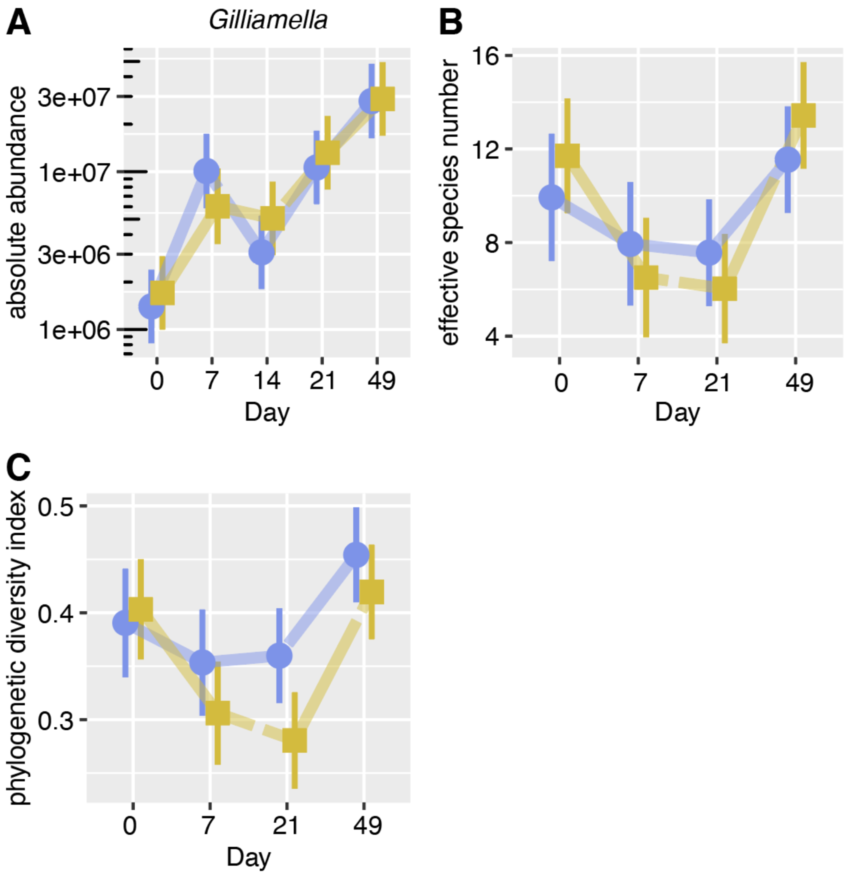

FIG S4. *Gilliamella* sp. (A) absolute abundance by sampling day (B) richness diversity estimate by Effective Species Number and (C) phylogenetic diversity estimate using Faith's phylogenetic index. No sampled points reflect a significant difference between treatment categories. Blue=Control; Yellow=Tylosin treatment.

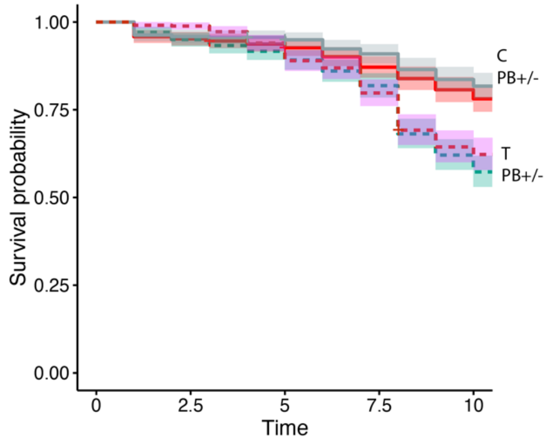

FIG S5. Survival probability over a ten day period of unchallenged (*Sm*-) Control and Tylan treated bees that were fed probiotic mix (PB+) or not (PB-). Within both groups, Control and Tylan, bees fed probiotic were not significantly different from bees not fed probiotic. Cox proportional hazards mixed effects model (by trial) n.s. = $P > 0.5$.

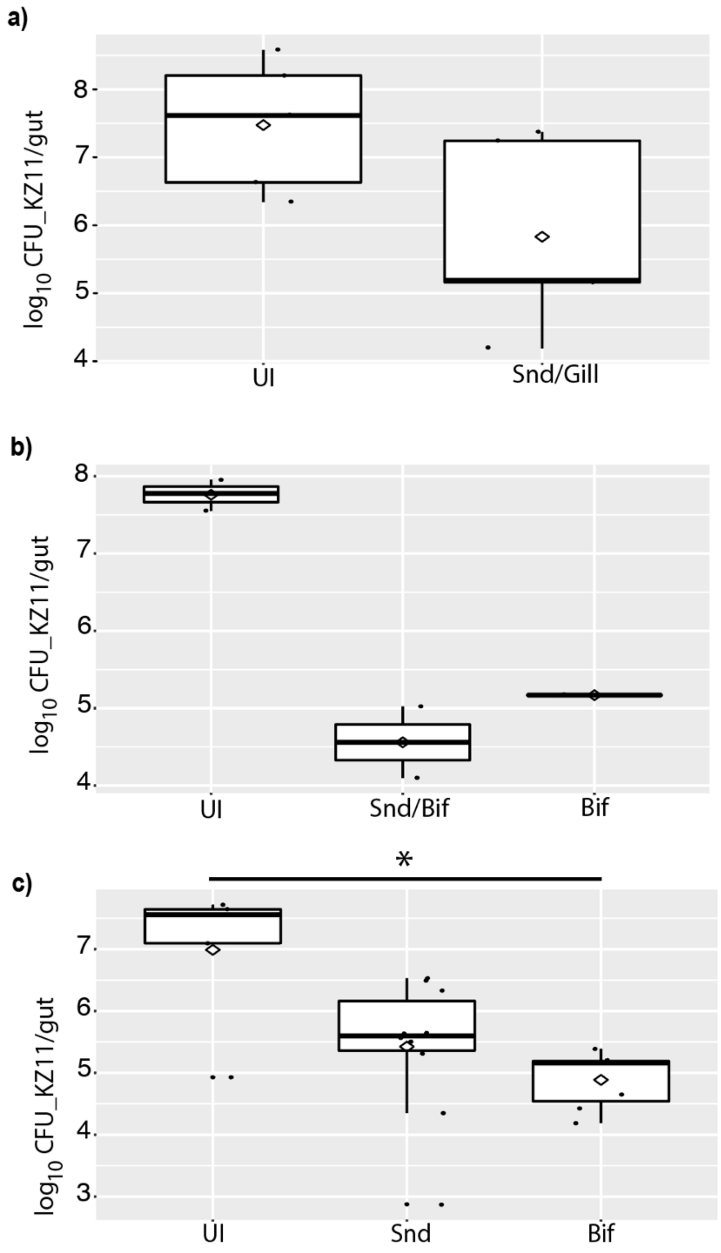

FIG. S6. Colony forming units (CFU) of the bacterial pathogen *Serratia marcescens* strain KZ11 (kan R) per plated bee gut. Recovered colonies of S. *marcescens* strain KZ11 (kan R) from uninoculated bees (UI), or those infected with (a) both S. alvi (wkB2) and G. apicola (wkB7). (b) S. alvi strain wkB2 (Snd) and *B. asteroides* strain LCep5 (Bif) or *Bif* alone. (c) Recovered S. *marcescens* colonies from UI guts or those colonized with Snd or Bif alone. (*, $p<0.05$, post-hoc pairwise comparisons using Tukey and Kramer (Nemenyi) test with Tukey distribution approximation for independent samples following Kruskal-Wallis).

SUPPLEMENTARY MATERIALS AND METHODS

High throughput sequencing of bacterial community

For community surveys of 16S V4 metabarcoded amplicons, the first PCR reaction amplified the diluted template in triplicate 20 µL reactions with Illumina-adapted 515F and 806R primers (See Table S2) (1) using 5 PRIME HotMasterMix (2.5X, Quantabio, MA, USA). Cycling conditions and Mastermix recipes may be viewed in Table S2). These 3 reactions were pooled, examined on a 2% agarose then purified with 0.8x HighPrep™ PCR magnetic beads (MAGBIO®, MD, USA). The cleaned product was diluted to a final volume of 52.5 µL. The second PCR reaction attached Illumina 8 bp Nextera style dual-indexed barcodes to 1 µL of pooled PCR 1 product and was performed in 25 µL single reactions using a unique combination of N7XX and S5XX. These reactions were cleaned with magnetic beads, resuspended in 27.5 µL molecular grade water and quantified on a plate reader using the Accublue broad range dsDNA quantitation kit (Biotium, Fremont, CA, USA). Equimolar amounts of each amplification within each sampling interval were pooled, combined with 5% phiX DNA and sequenced on an Illumina iSeq instrument at 2 x 150 reads.

For taxon-specific single copy gene metabarcoding, we used several lineage-specific targets: for *Snodgrassella* we used *minD* (2), for *Gilliamella rimM* (3), and for *Bifidobacterium* we used *groEL* (4). We amplified each one of these gene targets in a single 25 ul reaction with Accustart 2 mastermix (Quanta Bio, MA, USA). Table S2 lists cycling conditions and master mix recipes. We decided to use single reactions for these targets based on a recent publication that showed the triplicate pooling strategy is unnecessary for the reduction of amplification bias (5). Additionally, the 14-day time point was omitted from this portion. We screened, cleaned, barcoded and pooled the samples with the same strategy as with the 16S rRNA gene

metabarcoding portion except with the Accustart enzyme. We repeated any failed reactions with less dilute nucleic acid. Gene target amplifications of samples that were left out of the study, either due to lack of amplification of one or more genes or low read abundance during analysis are recorded in Table S4. We sent the pooled library to Psomagen Corp (MD, USA) for sequencing via a miSeq 2x300 run (Illumina, CA, USA). Demultiplexed read data was returned to us for subsequent analysis.

Primer sequences and amplification protocols are listed in Table S2.

Processing and analysis of high throughput reads

Demultiplexing of the iSeq 16S rRNA gene V4 metabarcode reads on the basis of barcode sequences was performed onboard the instrument by the iSeq software (Local Run Manager, Generate FASTQ Analysis Module 2.0). We then processed these split reads with QIIME 2 version 2019.10 (6). Forward reads were used alone because not enough overlap existed between paired end sequences to assemble them. Primer and adapter sequences were removed with the *cutadapt* plugin (7). Then they were truncated to 120 base pairs, filtered, denoised, and chimeric reads were removed using the *Deblur* plugin (8). Taxonomy was assigned to amplicon sequence variants (ASVs) using the SILVA 132-99-515-806-nb database with the *feature-classifier* plugin (9). We inspected ASVs and removed unassigned, mitochondrial, chloroplast, and singleton reads with the f*eature-table* plugin (10). We used the *align-to-tree-mafft-fasttree* phylogenetic tree building function to reconstruct a phylogenetic tree to use with diversity estimation models (11). For the visualization of composition within bee samples we combined ASVs within species groups and used a BLAST based binning strategy for the more complex lineages like *Lacobacillus* (made up of the bee specific clades of Firm-4 and

Firm-5 as well as environmental Firmicutes). Taxonomic barplots were produced within R with ggplot2 (12) of both relative abundance and absolute abundance (Figure S1a-b).

For the 16S rRNA gene metabarcoded reads, alpha diversity was first examined by performing 10 subsampling iterations at every 100 reads at each sampling interval. We used a uniform depth of 1000 reads per sample was decided upon, as this depth preserved a high number of samples for examination and showed saturation of alpha diversity. We realize that there is much debate currently as to the efficacy of subsampling in the practice of diversity estimation. Some argue that subsampling underestimates the true diversity of communities (13) while others argue that not subsampling may erroneously inflate the false discovery rate in examined datasets (14). We used subsampling mainly to remain consistent and maintain comparability with previous studies from our laboratory (2, 15–17). We looked at two main measures of alpha diversity in an effort to decipher the scope of changes that may have occurred with treatment and subsequent recovery. We examined species richness by obtaining the Shannon index (which factors both richness and evenness) for each sample and then using this to calculate the Effective Species Number (18) and also by using Faith's phylogenetic diversity metric (19) which looks at richness across phylogenetic lineages. We assessed differences in Alpha diversity between treatment bacteria between treatment groups at each sampling point by using linear mixed-effects models between control and treatment bees or hives, respectively. Treatment and sampling time were considered as fixed effects, and bees or bees nested within hives. Mixed models were fitted using the R package "lme4" (20) and followed by *post hoc* tests using the R package "emmeans" (21).

For the taxon-specific single copy gene method, we used a similar set of techniques as with the 16S rRNA gene V4 metabarcoding portion. However, by using longer paired end reads (2x300) we were able to assemble them into longer high-quality sequences with the *vsearch/join-pairs* plugin for Qiime2. These assembled sequences were then trimmed to the longest high-quality length that yielded the largest pool of total reads for each gene target. We also chose trim lengths which were within the sequenced portion of enough GenBank deposited sequences to allow for some confidence in phylogenetic tree inference and strain level taxonomic assignment. The trim length for *minD* (*Snodgrassella*) was 510 bp for *rimM* (*Gilliamella*) 295 bp and for *groEL* (*Bifidobacterium*) 420 bp. To perform these strain-level taxonomic analyses, we had to first construct databases and train classifiers for each gene target of deposited variants that were within a range of similarity to one another (see Table S4 for accession numbers) ~99.9 – 83%. These variants were retrieved, trimmed to the region of comparison, aligned and then used to train classifiers with the *feature-classifier/fit-classifier-naive-bayes* plugin in Qiime2. Alpha and Beta diversity analyses were then performed in the same manner as with the 16S rRNA gene V4 portion though subsampling levels were calculated per gene target based on read depth and saturation (*minD* = 400 reads, *rimM* = 1000 reads, *groEL*_Bifido = 600 reads). The analysis of strain-level differences focused on alpha and beta diversity using the above-mentioned statistical methods.

Beta diversity was examined by generating weighted UniFrac PCoA plots at each sampling point (22). Pairwise differences between the treatment groups were compared by using PERMANOVA (23) with 999 permutations. In order to examine differential abundances in taxonomic groups that may be driving differences in Beta diversity we employed the ANCOM compositional analysis tool (24) in Qiime2. In order to use ANCOM we first collapsed each

timepoint's taxonomic tables at level 5 and added a pseudocount before applying the tool. Differences flagged by ANCOM were further validated by statistically comparing absolute abundance (explained below) for each lineage between treatment groups at each sampling point using the generalized linear mixed-effects models (the *glm* model from the *lme4* package assuming poisson distribution, constructed and tested as above). Plots of these comparisons were generated in R with ggplot2 using scripts adapted from those developed by Erick V. S. Motta which are available at https://github.com/erickmotta/aem-2020 (16).

qPCR for 16S rRNA gene copy abundance

We used the techniques outlined in previous studies (17, 25) for absolute SYBR green qPCR quantitation of total 16S rRNA gene copies with the 27F/355R (See Table SI2) (26) Universal 16S primer set along with a serially diluted plasmid-based standard.

Resultant 16S rRNA gene copy counts were corrected for dilution and then the estimated absolute abundance for each bacterial species was calculated by multiplying the total number of 16S rRNA gene copies obtained by qPCR by the percent relative abundance of each species, adjusting based on genomic 16S rRNA gene copy number, as in (27).

We compared the absolute number of each lineage and total bacteria between treatment groups at each sampling point by using generalized linear mixed-effects models assuming Poisson distribution between control and treatment bees or hives, respectively and modelled as previously described.

REFERENCES

1. Wang Y, Qian PY. 2009. Conservative fragments in bacterial 16S rRNA genes and primer design for 16S ribosomal DNA amplicons in metagenomic studies. PLoS One 4.
2. Powell JE, Ratnayeke N, Moran NA. 2016. Strain diversity and host specificity in a specialized gut symbiont of honeybees and bumblebees. Mol Ecol 25:4461–4471.
3. Raymann K, Bobay L-M, Moran NA. 2017. Antibiotics reduce genetic diversity of core species in the honeybee gut microbiome 26:2057–2066.
4. Hu L, Lu W, Wang L, Pan M, Zhang H, Zhao J, Chen W. 2017. Assessment of Bifidobacterium species using groEL gene on the basis of illumina miseq high-throughput sequencing. Genes (Basel) 8.
5. Marotz C, Sharma A, Humphrey G, Gottel N, Daum C, Gilbert JA, Eloe-Fadrosh E, Knight R. 2019. Triplicate PCR reactions for 16S rRNA gene amplicon sequencing are unnecessary. Biotechniques 67:29–32.
6. Bolyen E, Rideout JR, Dillon MR, Bokulich NA, Abnet CC, Al-Ghalith GA, Alexander H, Alm EJ, Arumugam M, Asnicar F, Bai Y, Bisanz JE, Bittinger K, Brejnrod A, Brislawn CJ, Brown CT, Callahan BJ, Caraballo-Rodríguez AM, Chase J, Cope EK, Da Silva R, Diener C, Dorrestein PC, Douglas GM, Durall DM, Duvallet C, Edwardson CF, Ernst M, Estaki M, Fouquier J, Gauglitz JM, Gibbons SM, Gibson DL, Gonzalez A, Gorlick K, Guo J, Hillmann B, Holmes S, Holste H, Huttenhower C, Huttley GA, Janssen S, Jarmusch AK, Jiang L, Kaehler BD, Kang K Bin, Keefe CR, Keim P, Kelley ST, Knights D, Koester I, Kosciolek T, Kreps J, Langille MGI, Lee J, Ley R, Liu YX, Loftfield E, Lozupone C, Maher M, Marotz C, Martin BD, McDonald D, McIver LJ, Melnik A V., Metcalf JL, Morgan SC, Morton JT, Naimey AT, Navas-Molina JA, Nothias LF, Orchanian SB, Pearson T, Peoples SL, Petras D, Preuss ML, Pruesse E, Rasmussen LB, Rivers A, Robeson MS, Rosenthal P, Segata N, Shaffer M, Shiffer A, Sinha R, Song SJ, Spear JR, Swafford AD, Thompson LR, Torres PJ, Trinh P, Tripathi A, Turnbaugh PJ, Ul-Hasan S, van der Hooft JJJ, Vargas F, Vázquez-Baeza Y, Vogtmann E, von Hippel M, Walters W, Wan Y, Wang M, Warren J, Weber KC, Williamson CHD, Willis AD, Xu ZZ, Zaneveld JR, Zhang Y, Zhu Q, Knight R, Caporaso JG. 2019. Reproducible, interactive, scalable and extensible microbiome data science using QIIME 2. Nat Biotechnol 37:852–857.
7. Martin M. 2011. Cutadapt Removes Adapter Sequences From High-Throughput Sequencing Reads. EMBnet.journal 17:10–12.
8. Amir A, Daniel M, Navas-Molina J, Kopylova E, Morton J, Xu ZZ, Eric K, Thompson L, Hyde E, Gonzalez A, Knight R. 2017. Deblur Rapidly Resolves Single Nucleotide Community Sequence Patterns. Am Soc Microbiol 2:1–7.
9. Bokulich NA, Kaehler BD, Rideout JR, Dillon M, Bolyen E, Knight R, Huttley GA, Gregory Caporaso J. 2018. Optimizing taxonomic classification of marker-gene amplicon sequences with QIIME 2's q2-feature-classifier plugin. Microbiome 6:1–17.
10. Estaki M, Jiang L, Bokulich NA, McDonald D, González A, Kosciolek T, Martino C, Zhu Q, Birmingham A, Vázquez-Baeza Y, Dillon MR, Bolyen E, Caporaso JG, Knight R. 2020. QIIME 2 Enables Comprehensive End-to-End Analysis of Diverse Microbiome Data and Comparative Studies with Publicly Available Data. Curr Protoc Bioinforma 70:1–46.
11. Katoh K, Misawa K, Kuma KI, Miyata T. 2002. MAFFT: A novel method for rapid multiple sequence alignment based on fast Fourier transform. Nucleic Acids Res 30:3059–3066.

12. Wickham H. 2009. ggplot2 Elegant Graphics for Data Analysis. springer-Verlag, New York, USA.
13. McMurdie PJ, Holmes S. 2014. Waste Not, Want Not: Why Rarefying Microbiome Data Is Inadmissible. PLoS Comput Biol 10.
14. Weiss S, Xu ZZ, Peddada S, Amir A, Bittinger K, Gonzalez A, Lozupone C, Zaneveld JR, Vázquez-Baeza Y, Birmingham A, Hyde ER, Knight R. 2017. Normalization and microbial differential abundance strategies depend upon data characteristics. Microbiome 5:1–18.
15. Motta EVS, Raymann K, Moran NA. 2018. Glyphosate perturbs the gut microbiota of honey bees. Proc Natl Acad Sci U S A 115:10305–10310.
16. Motta EVS, Mak M, De Jong TK, Powell JE, O'Donnell A, Suhr KJ, Riddington IM, Moran NA. 2020. Oral or topical exposure to glyphosate in herbicide formulation impacts the gut microbiota and survival rates of honey bees. Appl Environ Microbiol 86:1–21.
17. Powell JE, Martinson VG, Urban-Mead K, Moran NA. 2014. Routes of acquisition of the gut microbiota of the honey bee Apis mellifera. Appl Environ Microbiol 80:7378–7387.
18. Jost L. 2006. Entropy AndDiversity. Oikos 2.
19. Faith DP, Baker AM. 2006. Phylogenetic diversity (PD) and biodiversity conservation: some bioinformatics challenges. Evol Bioinform Online 2:121–128.
20. Bates D, Mächler M, Bolker BM, Walker SC. 2015. Fitting linear mixed-effects models using lme4. J Stat Softw 67.
21. Lenth R, Singmann H, Love J, Buerkner P HM. 2019. emmeans: estimated marginal means, aka least-squares means.
22. Lozupone C, Lladser ME, Knights D, Stombaugh J, Knight R. 2011. UniFrac: An effective distance metric for microbial community comparison. ISME J 5:169–172.
23. Anderson MJ. 2001. A new method for non-parametric multivariate analysis of variance. Austral Ecol 26:32–46.
24. Mandal S, Van Treuren W, White RA, Eggesbø M, Knight R, Peddada SD. 2015. Analysis of composition of microbiomes: a novel method for studying microbial composition. Microb Ecol Heal Dis 26:1–7.
25. Martinson VG, Moy J, Moran NA. 2012. Establishment of characteristic gut bacteria during development of the honeybee worker. Appl Environ Microbiol 78:2830–2840.
26. Castillo M, Martín-Orúe SM, Manzanilla EG, Badiola I, Martín M, Gasa J. 2006. Quantification of total bacteria, enterobacteria and lactobacilli populations in pig digesta by real-time PCR. Vet Microbiol 114:165–170.
27. Raymann K, Shaffer Z, Moran NA. 2017. Antibiotic exposure perturbs the gut microbiota and elevates mortality in honeybees. PLoS Biol 15:e2001861.

Table S1. Sample collection information.

ID	Treatment	Description	Hive	Group	Collection#	Date Collecte	Day	LatLong
C3H1SE1	Pretreat	Control	1SE	1SE0	3	8/1/18	0	30.1114998_-98.0212251
C3H1SE2	Pretreat	Control	1SE	1SE0	3	8/1/18	0	30.1114998_-98.0212251
C3H1SE3	Pretreat	Control	1SE	1SE0	3	8/1/18	0	30.1114998_-98.0212251
C3H1SE4	Pretreat	Control	1SE	1SE0	3	8/1/18	0	30.1114998_-98.0212251
C3H1SE5	Pretreat	Control	1SE	1SE0	3	8/1/18	0	30.1114998_-98.0212251
C3H1SE8	Pretreat	Control	1SE	1SE0	3	8/1/18	0	30.1114998_-98.0212251
C3H1SE9	Pretreat	Control	1SE	1SE0	3	8/1/18	0	30.1114998_-98.0212251
C3H1SE10	Pretreat	Control	1SE	1SE0	3	8/1/18	0	30.1114998_-98.0212251
C3H30BW1	Pretreat	Control	30BW	30BW0	3	8/1/18	0	30.1114998_-98.0212251
C3H30BW2	Pretreat	Control	30BW	30BW0	3	8/1/18	0	30.1114998_-98.0212251
C3H30BW3	Pretreat	Control	30BW	30BW0	3	8/1/18	0	30.1114998_-98.0212251
C3H30BW4	Pretreat	Control	30BW	30BW0	3	8/1/18	0	30.1114998_-98.0212251
C3H30BW5	Pretreat	Control	30BW	30BW0	3	8/1/18	0	30.1114998_-98.0212251
C3H30BW7	Pretreat	Control	30BW	30BW0	3	8/1/18	0	30.1114998_-98.0212251
C3H30BW8	Pretreat	Control	30BW	30BW0	3	8/1/18	0	30.1114998_-98.0212251
C3H30BW9	Pretreat	Control	30BW	30BW0	3	8/1/18	0	30.1114998_-98.0212251
C3H4SE1	Pretreat	Control	4SE	4SE0	3	8/1/18	0	30.1114998_-98.0212251
C3H4SE2	Pretreat	Control	4SE	4SE0	3	8/1/18	0	30.1114998_-98.0212251
C3H4SE4	Pretreat	Control	4SE	4SE0	3	8/1/18	0	30.1114998_-98.0212251
C3H4SE7	Pretreat	Control	4SE	4SE0	3	8/1/18	0	30.1114998_-98.0212251
C3H4SE8	Pretreat	Control	4SE	4SE0	3	8/1/18	0	30.1114998_-98.0212251
C3H4SE9	Pretreat	Control	4SE	4SE0	3	8/1/18	0	30.1114998_-98.0212251
C3H4SE10	Pretreat	Control	4SE	4SE0	3	8/1/18	0	30.1114998_-98.0212251
C3H4SE11	Pretreat	Control	4SE	4SE0	3	8/1/18	0	30.1114998_-98.0212251
C3H5SE1	Pretreat	Control	5SE	5SE0	3	8/1/18	0	30.1114998_-98.0212251
C3H5SE2	Pretreat	Control	5SE	5SE0	3	8/1/18	0	30.1114998_-98.0212251
C3H5SE3	Pretreat	Control	5SE	5SE0	3	8/1/18	0	30.1114998_-98.0212251
C3H5SE4	Pretreat	Control	5SE	5SE0	3	8/1/18	0	30.1114998_-98.0212251
C3H5SE5	Pretreat	Control	5SE	5SE0	3	8/1/18	0	30.1114998_-98.0212251
C3H5SE7	Pretreat	Control	5SE	5SE0	3	8/1/18	0	30.1114998_-98.0212251
C3H5SE8	Pretreat	Control	5SE	5SE0	3	8/1/18	0	30.1114998_-98.0212251
C3H5SE9	Pretreat	Control	5SE	5SE0	3	8/1/18	0	30.1114998_-98.0212251
C3H8SE1	Pretreat	Control	8SE	8SE0	3	8/1/18	0	30.1114998_-98.0212251
C3H8SE2	Pretreat	Control	8SE	8SE0	3	8/1/18	0	30.1114998_-98.0212251
C3H8SE3	Pretreat	Control	8SE	8SE0	3	8/1/18	0	30.1114998_-98.0212251
C3H8SE4	Pretreat	Control	8SE	8SE0	3	8/1/18	0	30.1114998_-98.0212251
C3H8SE5	Pretreat	Control	8SE	8SE0	3	8/1/18	0	30.1114998_-98.0212251
C3H8SE7	Pretreat	Control	8SE	8SE0	3	8/1/18	0	30.1114998_-98.0212251
C3H8SE8	Pretreat	Control	8SE	8SE0	3	8/1/18	0	30.1114998_-98.0212251
C3H8SE9	Pretreat	Control	8SE	8SE0	3	8/1/18	0	30.1114998_-98.0212251
C3H11SE1	Pretreat	Tylosin	11SE	11SE0	3	8/1/18	0	30.1114998_-98.0212251
C3H11SE2	Pretreat	Tylosin	11SE	11SE0	3	8/1/18	0	30.1114998_-98.0212251
C3H11SE4	Pretreat	Tylosin	11SE	11SE0	3	8/1/18	0	30.1114998_-98.0212251
C3H11SE5	Pretreat	Tylosin	11SE	11SE0	3	8/1/18	0	30.1114998_-98.0212251
C3H11SE7	Pretreat	Tylosin	11SE	11SE0	3	8/1/18	0	30.1114998_-98.0212251
C3H11SE8	Pretreat	Tylosin	11SE	11SE0	3	8/1/18	0	30.1114998_-98.0212251
C3H11SE9	Pretreat	Tylosin	11SE	11SE0	3	8/1/18	0	30.1114998_-98.0212251
C3H11SE10	Pretreat	Tylosin	11SE	11SE0	3	8/1/18	0	30.1114998_-98.0212251
C3H12SE1	Pretreat	Tylosin	12SE	12SE0	3	8/1/18	0	30.1114998_-98.0212251
C3H12SE2	Pretreat	Tylosin	12SE	12SE0	3	8/1/18	0	30.1114998_-98.0212251
C3H12SE3	Pretreat	Tylosin	12SE	12SE0	3	8/1/18	0	30.1114998_-98.0212251
C3H12SE4	Pretreat	Tylosin	12SE	12SE0	3	8/1/18	0	30.1114998_-98.0212251
C3H12SE5	Pretreat	Tylosin	12SE	12SE0	3	8/1/18	0	30.1114998_-98.0212251
C3H12SE7	Pretreat	Tylosin	12SE	12SE0	3	8/1/18	0	30.1114998_-98.0212251
C3H12SE8	Pretreat	Tylosin	12SE	12SE0	3	8/1/18	0	30.1114998_-98.0212251
C3H12SE9	Pretreat	Tylosin	12SE	12SE0	3	8/1/18	0	30.1114998_-98.0212251
C3H13BW1	Pretreat	Tylosin	13BW	13BW0	3	8/1/18	0	30.1114998_-98.0212251
C3H13BW2	Pretreat	Tylosin	13BW	13BW0	3	8/1/18	0	30.1114998_-98.0212251
C3H13BW4	Pretreat	Tylosin	13BW	13BW0	3	8/1/18	0	30.1114998_-98.0212251
C3H13BW5	Pretreat	Tylosin	13BW	13BW0	3	8/1/18	0	30.1114998_-98.0212251
C3H13BW7	Pretreat	Tylosin	13BW	13BW0	3	8/1/18	0	30.1114998_-98.0212251
C3H13BW8	Pretreat	Tylosin	13BW	13BW0	3	8/1/18	0	30.1114998_-98.0212251
C3H13BW9	Pretreat	Tylosin	13BW	13BW0	3	8/1/18	0	30.1114998_-98.0212251
C3H13BW10	Pretreat	Tylosin	13BW	13BW0	3	8/1/18	0	30.1114998_-98.0212251
C3H14BW1	Pretreat	Tylosin	14BW	14BW0	3	8/1/18	0	30.1114998_-98.0212251

C3H14BW2	Pretreat	Tylosin	14BW	14BW0	3	8/1/18	0	30.1114998_-98.0212251
C3H14BW3	Pretreat	Tylosin	14BW	14BW0	3	8/1/18	0	30.1114998_-98.0212251
C3H14BW4	Pretreat	Tylosin	14BW	14BW0	3	8/1/18	0	30.1114998_-98.0212251
C3H14BW5	Pretreat	Tylosin	14BW	14BW0	3	8/1/18	0	30.1114998_-98.0212251
C3H14BW7	Pretreat	Tylosin	14BW	14BW0	3	8/1/18	0	30.1114998_-98.0212251
C3H14BW8	Pretreat	Tylosin	14BW	14BW0	3	8/1/18	0	30.1114998_-98.0212251
C3H14BW9	Pretreat	Tylosin	14BW	14BW0	3	8/1/18	0	30.1114998_-98.0212251
C3H15SE1	Pretreat	Tylosin	15SE	15SE0	3	8/1/18	0	30.1114998_-98.0212251
C3H15SE2	Pretreat	Tylosin	15SE	15SE0	3	8/1/18	0	30.1114998_-98.0212251
C3H15SE3	Pretreat	Tylosin	15SE	15SE0	3	8/1/18	0	30.1114998_-98.0212251
C3H15SE4	Pretreat	Tylosin	15SE	15SE0	3	8/1/18	0	30.1114998_-98.0212251
C3H15SE5	Pretreat	Tylosin	15SE	15SE0	3	8/1/18	0	30.1114998_-98.0212251
C3H15SE7	Pretreat	Tylosin	15SE	15SE0	3	8/1/18	0	30.1114998_-98.0212251
C3H15SE8	Pretreat	Tylosin	15SE	15SE0	3	8/1/18	0	30.1114998_-98.0212251
C3H15SE9	Pretreat	Tylosin	15SE	15SE0	3	8/1/18	0	30.1114998_-98.0212251
C4H1SE1	post1wk	Control	1SE	1SE7	4	8/8/18	7	30.1114998_-98.0212251
C4H1SE2	post1wk	Control	1SE	1SE7	4	8/8/18	7	30.1114998_-98.0212251
C4H1SE3	post1wk	Control	1SE	1SE7	4	8/8/18	7	30.1114998_-98.0212251
C4H1SE4	post1wk	Control	1SE	1SE7	4	8/8/18	7	30.1114998_-98.0212251
C4H1SE5	post1wk	Control	1SE	1SE7	4	8/8/18	7	30.1114998_-98.0212251
C4H1SE6	post1wk	Control	1SE	1SE7	4	8/8/18	7	30.1114998_-98.0212251
C4H1SE7	post1wk	Control	1SE	1SE7	4	8/8/18	7	30.1114998_-98.0212251
C4H1SE8	post1wk	Control	1SE	1SE7	4	8/8/18	7	30.1114998_-98.0212251
C4H30BW2	post1wk	Control	30BW	30BW7	4	8/8/18	7	30.1114998_-98.0212251
C4H30BW3	post1wk	Control	30BW	30BW7	4	8/8/18	7	30.1114998_-98.0212251
C4H30BW4	post1wk	Control	30BW	30BW7	4	8/8/18	7	30.1114998_-98.0212251
C4H30BW5	post1wk	Control	30BW	30BW7	4	8/8/18	7	30.1114998_-98.0212251
C4H30BW6	post1wk	Control	30BW	30BW7	4	8/8/18	7	30.1114998_-98.0212251
C4H30BW7	post1wk	Control	30BW	30BW7	4	8/8/18	7	30.1114998_-98.0212251
C4H30BW8	post1wk	Control	30BW	30BW7	4	8/8/18	7	30.1114998_-98.0212251
C4H4SE1	post1wk	Control	4SE	4SE7	4	8/8/18	7	30.1114998_-98.0212251
C4H4SE2	post1wk	Control	4SE	4SE7	4	8/8/18	7	30.1114998_-98.0212251
C4H4SE3	post1wk	Control	4SE	4SE7	4	8/8/18	7	30.1114998_-98.0212251
C4H4SE4	post1wk	Control	4SE	4SE7	4	8/8/18	7	30.1114998_-98.0212251
C4H4SE5	post1wk	Control	4SE	4SE7	4	8/8/18	7	30.1114998_-98.0212251
C4H4SE6	post1wk	Control	4SE	4SE7	4	8/8/18	7	30.1114998_-98.0212251
C4H4SE7	post1wk	Control	4SE	4SE7	4	8/8/18	7	30.1114998_-98.0212251
C4H4SE8	post1wk	Control	4SE	4SE7	4	8/8/18	7	30.1114998_-98.0212251
C4H5SE1	post1wk	Control	5SE	5SE7	4	8/8/18	7	30.1114998_-98.0212251
C4H5SE2	post1wk	Control	5SE	5SE7	4	8/8/18	7	30.1114998_-98.0212251
C4H5SE3	post1wk	Control	5SE	5SE7	4	8/8/18	7	30.1114998_-98.0212251
C4H5SE4	post1wk	Control	5SE	5SE7	4	8/8/18	7	30.1114998_-98.0212251
C4H5SE5	post1wk	Control	5SE	5SE7	4	8/8/18	7	30.1114998_-98.0212251
C4H5SE6	post1wk	Control	5SE	5SE7	4	8/8/18	7	30.1114998_-98.0212251
C4H5SE7	post1wk	Control	5SE	5SE7	4	8/8/18	7	30.1114998_-98.0212251
C4H5SE8	post1wk	Control	5SE	5SE7	4	8/8/18	7	30.1114998_-98.0212251
C4H8SE1	post1wk	Control	8SE	8SE7	4	8/8/18	7	30.1114998_-98.0212251
C4H8SE2	post1wk	Control	8SE	8SE7	4	8/8/18	7	30.1114998_-98.0212251
C4H8SE3	post1wk	Control	8SE	8SE7	4	8/8/18	7	30.1114998_-98.0212251
C4H8SE4	post1wk	Control	8SE	8SE7	4	8/8/18	7	30.1114998_-98.0212251
C4H8SE5	post1wk	Control	8SE	8SE7	4	8/8/18	7	30.1114998_-98.0212251
C4H8SE6	post1wk	Control	8SE	8SE7	4	8/8/18	7	30.1114998_-98.0212251
C4H8SE7	post1wk	Control	8SE	8SE7	4	8/8/18	7	30.1114998_-98.0212251
C4H8SE8	post1wk	Control	8SE	8SE7	4	8/8/18	7	30.1114998_-98.0212251
C4H11SE3	post1wk	Tylosin	11SE	11SE7	4	8/8/18	7	30.1114998_-98.0212251
C4H11SE4	post1wk	Tylosin	11SE	11SE7	4	8/8/18	7	30.1114998_-98.0212251
C4H11SE5	post1wk	Tylosin	11SE	11SE7	4	8/8/18	7	30.1114998_-98.0212251
C4H11SE6	post1wk	Tylosin	11SE	11SE7	4	8/8/18	7	30.1114998_-98.0212251
C4H11SE7	post1wk	Tylosin	11SE	11SE7	4	8/8/18	7	30.1114998_-98.0212251
C4H11SE8	post1wk	Tylosin	11SE	11SE7	4	8/8/18	7	30.1114998_-98.0212251
C4H12SE1	post1wk	Tylosin	12SE	12SE7	4	8/8/18	7	30.1114998_-98.0212251
C4H12SE2	post1wk	Tylosin	12SE	12SE7	4	8/8/18	7	30.1114998_-98.0212251
C4H12SE3	post1wk	Tylosin	12SE	12SE7	4	8/8/18	7	30.1114998_-98.0212251
C4H12SE4	post1wk	Tylosin	12SE	12SE7	4	8/8/18	7	30.1114998_-98.0212251
C4H12SE5	post1wk	Tylosin	12SE	12SE7	4	8/8/18	7	30.1114998_-98.0212251
C4H12SE6	post1wk	Tylosin	12SE	12SE7	4	8/8/18	7	30.1114998_-98.0212251
C4H12SE7	post1wk	Tylosin	12SE	12SE7	4	8/8/18	7	30.1114998_-98.0212251

C4H12SE8	post1wk	Tylosin	12SE	12SE7	4	8/8/18	7	30.1114998_-98.0212251
C4H13BW1	post1wk	Tylosin	13BW	13BW7	4	8/8/18	7	30.1114998_-98.0212251
C4H13BW2	post1wk	Tylosin	13BW	13BW7	4	8/8/18	7	30.1114998_-98.0212251
C4H13BW3	post1wk	Tylosin	13BW	13BW7	4	8/8/18	7	30.1114998_-98.0212251
C4H13BW4	post1wk	Tylosin	13BW	13BW7	4	8/8/18	7	30.1114998_-98.0212251
C4H13BW5	post1wk	Tylosin	13BW	13BW7	4	8/8/18	7	30.1114998_-98.0212251
C4H13BW6	post1wk	Tylosin	13BW	13BW7	4	8/8/18	7	30.1114998_-98.0212251
C4H13BW7	post1wk	Tylosin	13BW	13BW7	4	8/8/18	7	30.1114998_-98.0212251
C4H13BW8	post1wk	Tylosin	13BW	13BW7	4	8/8/18	7	30.1114998_-98.0212251
C4H14BW1	post1wk	Tylosin	14BW	14BW7	4	8/8/18	7	30.1114998_-98.0212251
C4H14BW2	post1wk	Tylosin	14BW	14BW7	4	8/8/18	7	30.1114998_-98.0212251
C4H14BW3	post1wk	Tylosin	14BW	14BW7	4	8/8/18	7	30.1114998_-98.0212251
C4H14BW4	post1wk	Tylosin	14BW	14BW7	4	8/8/18	7	30.1114998_-98.0212251
C4H14BW5	post1wk	Tylosin	14BW	14BW7	4	8/8/18	7	30.1114998_-98.0212251
C4H14BW6	post1wk	Tylosin	14BW	14BW7	4	8/8/18	7	30.1114998_-98.0212251
C4H14BW7	post1wk	Tylosin	14BW	14BW7	4	8/8/18	7	30.1114998_-98.0212251
C4H14BW8	post1wk	Tylosin	14BW	14BW7	4	8/8/18	7	30.1114998_-98.0212251
C4H15SE1	post1wk	Tylosin	15SE	15SE7	4	8/8/18	7	30.1114998_-98.0212251
C4H15SE2	post1wk	Tylosin	15SE	15SE7	4	8/8/18	7	30.1114998_-98.0212251
C4H15SE3	post1wk	Tylosin	15SE	15SE7	4	8/8/18	7	30.1114998_-98.0212251
C4H15SE4	post1wk	Tylosin	15SE	15SE7	4	8/8/18	7	30.1114998_-98.0212251
C4H15SE5	post1wk	Tylosin	15SE	15SE7	4	8/8/18	7	30.1114998_-98.0212251
C4H15SE6	post1wk	Tylosin	15SE	15SE7	4	8/8/18	7	30.1114998_-98.0212251
C4H15SE7	post1wk	Tylosin	15SE	15SE7	4	8/8/18	7	30.1114998_-98.0212251
C4H15SE8	post1wk	Tylosin	15SE	15SE7	4	8/8/18	7	30.1114998_-98.0212251
C5H1SE1	post14days	Control	1SE	1SE14	5	8/15/18	14	30.1114998_-98.0212251
C5H1SE2	post14days	Control	1SE	1SE14	5	8/15/18	14	30.1114998_-98.0212251
C5H1SE3	post14days	Control	1SE	1SE14	5	8/15/18	14	30.1114998_-98.0212251
C5H1SE4	post14days	Control	1SE	1SE14	5	8/15/18	14	30.1114998_-98.0212251
C5H1SE5	post14days	Control	1SE	1SE14	5	8/15/18	14	30.1114998_-98.0212251
C5H1SE6	post14days	Control	1SE	1SE14	5	8/15/18	14	30.1114998_-98.0212251
C5H1SE7	post14days	Control	1SE	1SE14	5	8/15/18	14	30.1114998_-98.0212251
C5H1SE8	post14days	Control	1SE	1SE14	5	8/15/18	14	30.1114998_-98.0212251
C5H30BW1	post14days	Control	30BW	30BW14	5	8/15/18	14	30.1114998_-98.0212251
C5H30BW2	post14days	Control	30BW	30BW14	5	8/15/18	14	30.1114998_-98.0212251
C5H30BW3	post14days	Control	30BW	30BW14	5	8/15/18	14	30.1114998_-98.0212251
C5H30BW4	post14days	Control	30BW	30BW14	5	8/15/18	14	30.1114998_-98.0212251
C5H30BW5	post14days	Control	30BW	30BW14	5	8/15/18	14	30.1114998_-98.0212251
C5H30BW6	post14days	Control	30BW	30BW14	5	8/15/18	14	30.1114998_-98.0212251
C5H30BW7	post14days	Control	30BW	30BW14	5	8/15/18	14	30.1114998_-98.0212251
C5H30BW8	post14days	Control	30BW	30BW14	5	8/15/18	14	30.1114998_-98.0212251
C5H4SE1	post14days	Control	4SE	4SE14	5	8/15/18	14	30.1114998_-98.0212251
C5H4SE2	post14days	Control	4SE	4SE14	5	8/15/18	14	30.1114998_-98.0212251
C5H4SE3	post14days	Control	4SE	4SE14	5	8/15/18	14	30.1114998_-98.0212251
C5H4SE4	post14days	Control	4SE	4SE14	5	8/15/18	14	30.1114998_-98.0212251
C5H4SE5	post14days	Control	4SE	4SE14	5	8/15/18	14	30.1114998_-98.0212251
C5H4SE6	post14days	Control	4SE	4SE14	5	8/15/18	14	30.1114998_-98.0212251
C5H4SE7	post14days	Control	4SE	4SE14	5	8/15/18	14	30.1114998_-98.0212251
C5H4SE8	post14days	Control	4SE	4SE14	5	8/15/18	14	30.1114998_-98.0212251
C5H5SE1	post14days	Control	5SE	5SE14	5	8/15/18	14	30.1114998_-98.0212251
C5H5SE2	post14days	Control	5SE	5SE14	5	8/15/18	14	30.1114998_-98.0212251
C5H5SE3	post14days	Control	5SE	5SE14	5	8/15/18	14	30.1114998_-98.0212251
C5H5SE4	post14days	Control	5SE	5SE14	5	8/15/18	14	30.1114998_-98.0212251
C5H5SE5	post14days	Control	5SE	5SE14	5	8/15/18	14	30.1114998_-98.0212251
C5H5SE6	post14days	Control	5SE	5SE14	5	8/15/18	14	30.1114998_-98.0212251
C5H5SE7	post14days	Control	5SE	5SE14	5	8/15/18	14	30.1114998_-98.0212251
C5H5SE8	post14days	Control	5SE	5SE14	5	8/15/18	14	30.1114998_-98.0212251
C5H8SE1	post14days	Control	8SE	8SE14	5	8/15/18	14	30.1114998_-98.0212251
C5H8SE2	post14days	Control	8SE	8SE14	5	8/15/18	14	30.1114998_-98.0212251
C5H8SE3	post14days	Control	8SE	8SE14	5	8/15/18	14	30.1114998_-98.0212251
C5H8SE4	post14days	Control	8SE	8SE14	5	8/15/18	14	30.1114998_-98.0212251
C5H8SE5	post14days	Control	8SE	8SE14	5	8/15/18	14	30.1114998_-98.0212251
C5H8SE6	post14days	Control	8SE	8SE14	5	8/15/18	14	30.1114998_-98.0212251
C5H8SE7	post14days	Control	8SE	8SE14	5	8/15/18	14	30.1114998_-98.0212251
C5H8SE8	post14days	Control	8SE	8SE14	5	8/15/18	14	30.1114998_-98.0212251
C5H11SE1	post14days	Tylosin	11SE	11SE14	5	8/15/18	14	30.1114998_-98.0212251
C5H11SE2	post14days	Tylosin	11SE	11SE14	5	8/15/18	14	30.1114998_-98.0212251

C5H11SE3	post14days	Tylosin	11SE	11SE14	5	8/15/18	14	30.1114998_-98.0212251
C5H11SE4	post14days	Tylosin	11SE	11SE14	5	8/15/18	14	30.1114998_-98.0212251
C5H11SE5	post14days	Tylosin	11SE	11SE14	5	8/15/18	14	30.1114998_-98.0212251
C5H11SE6	post14days	Tylosin	11SE	11SE14	5	8/15/18	14	30.1114998_-98.0212251
C5H11SE7	post14days	Tylosin	11SE	11SE14	5	8/15/18	14	30.1114998_-98.0212251
C5H11SE8	post14days	Tylosin	11SE	11SE14	5	8/15/18	14	30.1114998_-98.0212251
C5H12SE1	post14days	Tylosin	12SE	12SE14	5	8/15/18	14	30.1114998_-98.0212251
C5H12SE2	post14days	Tylosin	12SE	12SE14	5	8/15/18	14	30.1114998_-98.0212251
C5H12SE3	post14days	Tylosin	12SE	12SE14	5	8/15/18	14	30.1114998_-98.0212251
C5H12SE4	post14days	Tylosin	12SE	12SE14	5	8/15/18	14	30.1114998_-98.0212251
C5H12SE5	post14days	Tylosin	12SE	12SE14	5	8/15/18	14	30.1114998_-98.0212251
C5H12SE6	post14days	Tylosin	12SE	12SE14	5	8/15/18	14	30.1114998_-98.0212251
C5H12SE7	post14days	Tylosin	12SE	12SE14	5	8/15/18	14	30.1114998_-98.0212251
C5H12SE8	post14days	Tylosin	12SE	12SE14	5	8/15/18	14	30.1114998_-98.0212251
C5H13BW1	post14days	Tylosin	13BW	13BW14	5	8/15/18	14	30.1114998_-98.0212251
C5H13BW2	post14days	Tylosin	13BW	13BW14	5	8/15/18	14	30.1114998_-98.0212251
C5H13BW3	post14days	Tylosin	13BW	13BW14	5	8/15/18	14	30.1114998_-98.0212251
C5H13BW4	post14days	Tylosin	13BW	13BW14	5	8/15/18	14	30.1114998_-98.0212251
C5H13BW5	post14days	Tylosin	13BW	13BW14	5	8/15/18	14	30.1114998_-98.0212251
C5H13BW6	post14days	Tylosin	13BW	13BW14	5	8/15/18	14	30.1114998_-98.0212251
C5H13BW7	post14days	Tylosin	13BW	13BW14	5	8/15/18	14	30.1114998_-98.0212251
C5H13BW8	post14days	Tylosin	13BW	13BW14	5	8/15/18	14	30.1114998_-98.0212251
C5H14BW1	post14days	Tylosin	14BW	14BW14	5	8/15/18	14	30.1114998_-98.0212251
C5H14BW2	post14days	Tylosin	14BW	14BW14	5	8/15/18	14	30.1114998_-98.0212251
C5H14BW3	post14days	Tylosin	14BW	14BW14	5	8/15/18	14	30.1114998_-98.0212251
C5H14BW4	post14days	Tylosin	14BW	14BW14	5	8/15/18	14	30.1114998_-98.0212251
C5H14BW5	post14days	Tylosin	14BW	14BW14	5	8/15/18	14	30.1114998_-98.0212251
C5H14BW6	post14days	Tylosin	14BW	14BW14	5	8/15/18	14	30.1114998_-98.0212251
C5H14BW7	post14days	Tylosin	14BW	14BW14	5	8/15/18	14	30.1114998_-98.0212251
C5H14BW8	post14days	Tylosin	14BW	14BW14	5	8/15/18	14	30.1114998_-98.0212251
C5H15SE1	post14days	Tylosin	15SE	15SE14	5	8/15/18	14	30.1114998_-98.0212251
C5H15SE2	post14days	Tylosin	15SE	15SE14	5	8/15/18	14	30.1114998_-98.0212251
C5H15SE3	post14days	Tylosin	15SE	15SE14	5	8/15/18	14	30.1114998_-98.0212251
C5H15SE4	post14days	Tylosin	15SE	15SE14	5	8/15/18	14	30.1114998_-98.0212251
C5H15SE5	post14days	Tylosin	15SE	15SE14	5	8/15/18	14	30.1114998_-98.0212251
C5H15SE6	post14days	Tylosin	15SE	15SE14	5	8/15/18	14	30.1114998_-98.0212251
C5H15SE7	post14days	Tylosin	15SE	15SE14	5	8/15/18	14	30.1114998_-98.0212251
C5H15SE8	post14days	Tylosin	15SE	15SE14	5	8/15/18	14	30.1114998_-98.0212251
C6H1SE1	Post21days	Control	1SE	1SE21	6	8/22/18	21	30.1114998_-98.0212251
C6H1SE2	Post21days	Control	1SE	1SE21	6	8/22/18	21	30.1114998_-98.0212251
C6H1SE3	Post21days	Control	1SE	1SE21	6	8/22/18	21	30.1114998_-98.0212251
C6H1SE4	Post21days	Control	1SE	1SE21	6	8/22/18	21	30.1114998_-98.0212251
C6H1SE5	Post21days	Control	1SE	1SE21	6	8/22/18	21	30.1114998_-98.0212251
C6H1SE7	Post21days	Control	1SE	1SE21	6	8/22/18	21	30.1114998_-98.0212251
C6H1SE8	Post21days	Control	1SE	1SE21	6	8/22/18	21	30.1114998_-98.0212251
C6H1SE9	Post21days	Control	1SE	1SE21	6	8/22/18	21	30.1114998_-98.0212251
C6H30BW1	Post21days	Control	30BW	30BW21	6	8/22/18	21	30.1114998_-98.0212251
C6H30BW2	Post21days	Control	30BW	30BW21	6	8/22/18	21	30.1114998_-98.0212251
C6H30BW3	Post21days	Control	30BW	30BW21	6	8/22/18	21	30.1114998_-98.0212251
C6H30BW4	Post21days	Control	30BW	30BW21	6	8/22/18	21	30.1114998_-98.0212251
C6H30BW5	Post21days	Control	30BW	30BW21	6	8/22/18	21	30.1114998_-98.0212251
C6H30BW7	Post21days	Control	30BW	30BW21	6	8/22/18	21	30.1114998_-98.0212251
C6H30BW8	Post21days	Control	30BW	30BW21	6	8/22/18	21	30.1114998_-98.0212251
C6H30BW9	Post21days	Control	30BW	30BW21	6	8/22/18	21	30.1114998_-98.0212251
C6H4SE1	Post21days	Control	4SE	4SE21	6	8/22/18	21	30.1114998_-98.0212251
C6H4SE2	Post21days	Control	4SE	4SE21	6	8/22/18	21	30.1114998_-98.0212251
C6H4SE3	Post21days	Control	4SE	4SE21	6	8/22/18	21	30.1114998_-98.0212251
C6H4SE4	Post21days	Control	4SE	4SE21	6	8/22/18	21	30.1114998_-98.0212251
C6H4SE5	Post21days	Control	4SE	4SE21	6	8/22/18	21	30.1114998_-98.0212251
C6H4SE7	Post21days	Control	4SE	4SE21	6	8/22/18	21	30.1114998_-98.0212251
C6H4SE8	Post21days	Control	4SE	4SE21	6	8/22/18	21	30.1114998_-98.0212251
C6H4SE9	Post21days	Control	4SE	4SE21	6	8/22/18	21	30.1114998_-98.0212251
C6H5SE1	Post21days	Control	5SE	5SE21	6	8/22/18	21	30.1114998_-98.0212251
C6H5SE2	Post21days	Control	5SE	5SE21	6	8/22/18	21	30.1114998_-98.0212251
C6H5SE3	Post21days	Control	5SE	5SE21	6	8/22/18	21	30.1114998_-98.0212251
C6H5SE4	Post21days	Control	5SE	5SE21	6	8/22/18	21	30.1114998_-98.0212251
C6H5SE5	Post21days	Control	5SE	5SE21	6	8/22/18	21	30.1114998_-98.0212251

C6H5SE7	Post21days	Control	5SE	5SE21	6	8/22/18	21	30.1114998_-98.0212251
C6H5SE8	Post21days	Control	5SE	5SE21	6	8/22/18	21	30.1114998_-98.0212251
C6H5SE9	Post21days	Control	5SE	5SE21	6	8/22/18	21	30.1114998_-98.0212251
C6H8SE1	Post21days	Control	8SE	8SE21	6	8/22/18	21	30.1114998_-98.0212251
C6H8SE2	Post21days	Control	8SE	8SE21	6	8/22/18	21	30.1114998_-98.0212251
C6H8SE3	Post21days	Control	8SE	8SE21	6	8/22/18	21	30.1114998_-98.0212251
C6H8SE4	Post21days	Control	8SE	8SE21	6	8/22/18	21	30.1114998_-98.0212251
C6H8SE5	Post21days	Control	8SE	8SE21	6	8/22/18	21	30.1114998_-98.0212251
C6H8SE7	Post21days	Control	8SE	8SE21	6	8/22/18	21	30.1114998_-98.0212251
C6H8SE8	Post21days	Control	8SE	8SE21	6	8/22/18	21	30.1114998_-98.0212251
C6H8SE9	Post21days	Control	8SE	8SE21	6	8/22/18	21	30.1114998_-98.0212251
C6H11SE1	Post21days	Tylosin	11SE	11SE21	6	8/22/18	21	30.1114998_-98.0212251
C6H11SE2	Post21days	Tylosin	11SE	11SE21	6	8/22/18	21	30.1114998_-98.0212251
C6H11SE3	Post21days	Tylosin	11SE	11SE21	6	8/22/18	21	30.1114998_-98.0212251
C6H11SE4	Post21days	Tylosin	11SE	11SE21	6	8/22/18	21	30.1114998_-98.0212251
C6H11SE5	Post21days	Tylosin	11SE	11SE21	6	8/22/18	21	30.1114998_-98.0212251
C6H11SE7	Post21days	Tylosin	11SE	11SE21	6	8/22/18	21	30.1114998_-98.0212251
C6H11SE8	Post21days	Tylosin	11SE	11SE21	6	8/22/18	21	30.1114998_-98.0212251
C6H11SE9	Post21days	Tylosin	11SE	11SE21	6	8/22/18	21	30.1114998_-98.0212251
C6H12SE1	Post21days	Tylosin	12SE	12SE21	6	8/22/18	21	30.1114998_-98.0212251
C6H12SE2	Post21days	Tylosin	12SE	12SE21	6	8/22/18	21	30.1114998_-98.0212251
C6H12SE3	Post21days	Tylosin	12SE	12SE21	6	8/22/18	21	30.1114998_-98.0212251
C6H12SE4	Post21days	Tylosin	12SE	12SE21	6	8/22/18	21	30.1114998_-98.0212251
C6H12SE5	Post21days	Tylosin	12SE	12SE21	6	8/22/18	21	30.1114998_-98.0212251
C6H12SE7	Post21days	Tylosin	12SE	12SE21	6	8/22/18	21	30.1114998_-98.0212251
C6H12SE8	Post21days	Tylosin	12SE	12SE21	6	8/22/18	21	30.1114998_-98.0212251
C6H12SE9	Post21days	Tylosin	12SE	12SE21	6	8/22/18	21	30.1114998_-98.0212251
C6H13BW1	Post21days	Tylosin	13BW	13BW21	6	8/22/18	21	30.1114998_-98.0212251
C6H13BW2	Post21days	Tylosin	13BW	13BW21	6	8/22/18	21	30.1114998_-98.0212251
C6H13BW3	Post21days	Tylosin	13BW	13BW21	6	8/22/18	21	30.1114998_-98.0212251
C6H13BW4	Post21days	Tylosin	13BW	13BW21	6	8/22/18	21	30.1114998_-98.0212251
C6H13BW5	Post21days	Tylosin	13BW	13BW21	6	8/22/18	21	30.1114998_-98.0212251
C6H13BW7	Post21days	Tylosin	13BW	13BW21	6	8/22/18	21	30.1114998_-98.0212251
C6H13BW8	Post21days	Tylosin	13BW	13BW21	6	8/22/18	21	30.1114998_-98.0212251
C6H13BW9	Post21days	Tylosin	13BW	13BW21	6	8/22/18	21	30.1114998_-98.0212251
C6H14BW1	Post21days	Tylosin	14BW	14BW21	6	8/22/18	21	30.1114998_-98.0212251
C6H14BW2	Post21days	Tylosin	14BW	14BW21	6	8/22/18	21	30.1114998_-98.0212251
C6H14BW3	Post21days	Tylosin	14BW	14BW21	6	8/22/18	21	30.1114998_-98.0212251
C6H14BW4	Post21days	Tylosin	14BW	14BW21	6	8/22/18	21	30.1114998_-98.0212251
C6H14BW5	Post21days	Tylosin	14BW	14BW21	6	8/22/18	21	30.1114998_-98.0212251
C6H14BW7	Post21days	Tylosin	14BW	14BW21	6	8/22/18	21	30.1114998_-98.0212251
C6H14BW8	Post21days	Tylosin	14BW	14BW21	6	8/22/18	21	30.1114998_-98.0212251
C6H14BW9	Post21days	Tylosin	14BW	14BW21	6	8/22/18	21	30.1114998_-98.0212251
C6H15SE1	Post21days	Tylosin	15SE	15SE21	6	8/22/18	21	30.1114998_-98.0212251
C6H15SE2	Post21days	Tylosin	15SE	15SE21	6	8/22/18	21	30.1114998_-98.0212251
C6H15SE3	Post21days	Tylosin	15SE	15SE21	6	8/22/18	21	30.1114998_-98.0212251
C6H15SE4	Post21days	Tylosin	15SE	15SE21	6	8/22/18	21	30.1114998_-98.0212251
C6H15SE5	Post21days	Tylosin	15SE	15SE21	6	8/22/18	21	30.1114998_-98.0212251
C6H15SE7	Post21days	Tylosin	15SE	15SE21	6	8/22/18	21	30.1114998_-98.0212251
C6H15SE8	Post21days	Tylosin	15SE	15SE21	6	8/22/18	21	30.1114998_-98.0212251
C6H15SE9	Post21days	Tylosin	15SE	15SE21	6	8/22/18	21	30.1114998_-98.0212251
C8H1SE1	post1month	Control	1SE	1SE49	8	9/19/18	49	30.1114998_-98.0212251
C8H1SE2	post1month	Control	1SE	1SE49	8	9/19/18	49	30.1114998_-98.0212251
C8H1SE3	post1month	Control	1SE	1SE49	8	9/19/18	49	30.1114998_-98.0212251
C8H1SE4	post1month	Control	1SE	1SE49	8	9/19/18	49	30.1114998_-98.0212251
C8H1SE5	post1month	Control	1SE	1SE49	8	9/19/18	49	30.1114998_-98.0212251
C8H1SE6	post1month	Control	1SE	1SE49	8	9/19/18	49	30.1114998_-98.0212251
C8H1SE7	post1month	Control	1SE	1SE49	8	9/19/18	49	30.1114998_-98.0212251
C8H1SE8	post1month	Control	1SE	1SE49	8	9/19/18	49	30.1114998_-98.0212251
C8H30BW1	post1month	Control	30BW	30BW49	8	9/19/18	49	30.1114998_-98.0212251
C8H30BW2	post1month	Control	30BW	30BW49	8	9/19/18	49	30.1114998_-98.0212251
C8H30BW3	post1month	Control	30BW	30BW49	8	9/19/18	49	30.1114998_-98.0212251
C8H30BW5	post1month	Control	30BW	30BW49	8	9/19/18	49	30.1114998_-98.0212251
C8H30BW6	post1month	Control	30BW	30BW49	8	9/19/18	49	30.1114998_-98.0212251
C8H30BW7	post1month	Control	30BW	30BW49	8	9/19/18	49	30.1114998_-98.0212251
C8H30BW8	post1month	Control	30BW	30BW49	8	9/19/18	49	30.1114998_-98.0212251
C8H4SE1	post1month	Control	4SE	4SE49	8	9/19/18	49	30.1114998_-98.0212251

C8H4SE2	post1month	Control	4SE	4SE49	8	9/19/18	49	30.1114998_-98.0212251
C8H4SE3	post1month	Control	4SE	4SE49	8	9/19/18	49	30.1114998_-98.0212251
C8H4SE4	post1month	Control	4SE	4SE49	8	9/19/18	49	30.1114998_-98.0212251
C8H4SE5	post1month	Control	4SE	4SE49	8	9/19/18	49	30.1114998_-98.0212251
C8H4SE6	post1month	Control	4SE	4SE49	8	9/19/18	49	30.1114998_-98.0212251
C8H4SE7	post1month	Control	4SE	4SE49	8	9/19/18	49	30.1114998_-98.0212251
C8H4SE8	post1month	Control	4SE	4SE49	8	9/19/18	49	30.1114998_-98.0212251
C8H5SE1	post1month	Control	5SE	5SE49	8	9/19/18	49	30.1114998_-98.0212251
C8H5SE2	post1month	Control	5SE	5SE49	8	9/19/18	49	30.1114998_-98.0212251
C8H5SE3	post1month	Control	5SE	5SE49	8	9/19/18	49	30.1114998_-98.0212251
C8H5SE4	post1month	Control	5SE	5SE49	8	9/19/18	49	30.1114998_-98.0212251
C8H5SE5	post1month	Control	5SE	5SE49	8	9/19/18	49	30.1114998_-98.0212251
C8H5SE6	post1month	Control	5SE	5SE49	8	9/19/18	49	30.1114998_-98.0212251
C8H5SE7	post1month	Control	5SE	5SE49	8	9/19/18	49	30.1114998_-98.0212251
C8H5SE8	post1month	Control	5SE	5SE49	8	9/19/18	49	30.1114998_-98.0212251
C8H8SE1	post1month	Control	8SE	8SE49	8	9/19/18	49	30.1114998_-98.0212251
C8H8SE2	post1month	Control	8SE	8SE49	8	9/19/18	49	30.1114998_-98.0212251
C8H8SE3	post1month	Control	8SE	8SE49	8	9/19/18	49	30.1114998_-98.0212251
C8H8SE4	post1month	Control	8SE	8SE49	8	9/19/18	49	30.1114998_-98.0212251
C8H8SE5	post1month	Control	8SE	8SE49	8	9/19/18	49	30.1114998_-98.0212251
C8H8SE6	post1month	Control	8SE	8SE49	8	9/19/18	49	30.1114998_-98.0212251
C8H8SE7	post1month	Control	8SE	8SE49	8	9/19/18	49	30.1114998_-98.0212251
C8H8SE8	post1month	Control	8SE	8SE49	8	9/19/18	49	30.1114998_-98.0212251
C8H11SE1	post1month	Tylosin	11SE	11SE49	8	9/19/18	49	30.1114998_-98.0212251
C8H11SE2	post1month	Tylosin	11SE	11SE49	8	9/19/18	49	30.1114998_-98.0212251
C8H11SE3	post1month	Tylosin	11SE	11SE49	8	9/19/18	49	30.1114998_-98.0212251
C8H11SE4	post1month	Tylosin	11SE	11SE49	8	9/19/18	49	30.1114998_-98.0212251
C8H11SE5	post1month	Tylosin	11SE	11SE49	8	9/19/18	49	30.1114998_-98.0212251
C8H11SE6	post1month	Tylosin	11SE	11SE49	8	9/19/18	49	30.1114998_-98.0212251
C8H11SE7	post1month	Tylosin	11SE	11SE49	8	9/19/18	49	30.1114998_-98.0212251
C8H11SE8	post1month	Tylosin	11SE	11SE49	8	9/19/18	49	30.1114998_-98.0212251
C8H12SE1	post1month	Tylosin	12SE	12SE49	8	9/19/18	49	30.1114998_-98.0212251
C8H12SE2	post1month	Tylosin	12SE	12SE49	8	9/19/18	49	30.1114998_-98.0212251
C8H12SE3	post1month	Tylosin	12SE	12SE49	8	9/19/18	49	30.1114998_-98.0212251
C8H12SE4	post1month	Tylosin	12SE	12SE49	8	9/19/18	49	30.1114998_-98.0212251
C8H12SE5	post1month	Tylosin	12SE	12SE49	8	9/19/18	49	30.1114998_-98.0212251
C8H12SE6	post1month	Tylosin	12SE	12SE49	8	9/19/18	49	30.1114998_-98.0212251
C8H12SE7	post1month	Tylosin	12SE	12SE49	8	9/19/18	49	30.1114998_-98.0212251
C8H12SE8	post1month	Tylosin	12SE	12SE49	8	9/19/18	49	30.1114998_-98.0212251
C8H13BW1	post1month	Tylosin	13BW	13BW49	8	9/19/18	49	30.1114998_-98.0212251
C8H13BW2	post1month	Tylosin	13BW	13BW49	8	9/19/18	49	30.1114998_-98.0212251
C8H13BW3	post1month	Tylosin	13BW	13BW49	8	9/19/18	49	30.1114998_-98.0212251
C8H13BW4	post1month	Tylosin	13BW	13BW49	8	9/19/18	49	30.1114998_-98.0212251
C8H13BW5	post1month	Tylosin	13BW	13BW49	8	9/19/18	49	30.1114998_-98.0212251
C8H13BW6	post1month	Tylosin	13BW	13BW49	8	9/19/18	49	30.1114998_-98.0212251
C8H13BW7	post1month	Tylosin	13BW	13BW49	8	9/19/18	49	30.1114998_-98.0212251
C8H13BW8	post1month	Tylosin	13BW	13BW49	8	9/19/18	49	30.1114998_-98.0212251
C8H14BW1	post1month	Tylosin	14BW	14BW49	8	9/19/18	49	30.1114998_-98.0212251
C8H14BW2	post1month	Tylosin	14BW	14BW49	8	9/19/18	49	30.1114998_-98.0212251
C8H14BW3	post1month	Tylosin	14BW	14BW49	8	9/19/18	49	30.1114998_-98.0212251
C8H14BW4	post1month	Tylosin	14BW	14BW49	8	9/19/18	49	30.1114998_-98.0212251
C8H14BW5	post1month	Tylosin	14BW	14BW49	8	9/19/18	49	30.1114998_-98.0212251
C8H14BW6	post1month	Tylosin	14BW	14BW49	8	9/19/18	49	30.1114998_-98.0212251
C8H14BW7	post1month	Tylosin	14BW	14BW49	8	9/19/18	49	30.1114998_-98.0212251
C8H14BW8	post1month	Tylosin	14BW	14BW49	8	9/19/18	49	30.1114998_-98.0212251
C8H15SE1	post1month	Tylosin	15SE	15SE49	8	9/19/18	49	30.1114998_-98.0212251
C8H15SE2	post1month	Tylosin	15SE	15SE49	8	9/19/18	49	30.1114998_-98.0212251
C8H15SE3	post1month	Tylosin	15SE	15SE49	8	9/19/18	49	30.1114998_-98.0212251
C8H15SE4	post1month	Tylosin	15SE	15SE49	8	9/19/18	49	30.1114998_-98.0212251
C8H15SE5	post1month	Tylosin	15SE	15SE49	8	9/19/18	49	30.1114998_-98.0212251
C8H15SE6	post1month	Tylosin	15SE	15SE49	8	9/19/18	49	30.1114998_-98.0212251
C8H15SE7	post1month	Tylosin	15SE	15SE49	8	9/19/18	49	30.1114998_-98.0212251
C8H15SE8	post1month	Tylosin	15SE	15SE49	8	9/19/18	49	30.1114998_-98.0212251

Table S2. List of primers and PCR protocols.

Purpose	Forward		Reverse		Cycling Protocol			Chemistry	Reference/Notes
16S V4 PCR Round 1	Hyb515F_rRNA	(5'-TCGTCGGCAGCGTCAGATGTGTATAAGAGACAGGTGYCAGCMGCCGCGGTA -3')	Hyb806R_rRNA	(5'-GTCTCGTGGGCTCGGAGATGTGTATAAGAGACAGGGACTACHVGGGTWTCTAAT -3')	94°C	3 min	1x	PCR reactions were conducted in (3x) 20 µL reactions of: Mol bio grade water 10.2ul<br>Hyb515F_rRNA (10uM) 0.4 ul<br>Hyb806R_rRNA2 (10uM) 0.4 ul<br>5-PRIME HotMasterMix (2.5x) 8 uL<br>Template DNA 1ul (~10ng)	(1)
					94°C	45 sec	30x		
					50°C	60 sec			
					72°C	90 sec			
					72°C	10 min	1x		
					4°C	∞	hold		
minD (Snodgrassella single copy gene) PCR Round 1	RND1_MinDF1	(5‘-TCGTCGGCAGCGTCAGATGTGTATAAGAGACAGTGCGGTAATTGATTTTGATG--3‘)	RND1_MinDR2wbl2	(5‘-GTCTCGTGGGCTCGGAGATGTGTATAAGAGACAGRCGYAAAATATCCTGAATATC-3‘)	94°C	3 min	1x	PCR reactions were conducted in 20 µL reactions of: Mol bio grade water 10.2ul<br>Forward (10uM) 0.4 ul<br>Reverse (10uM) 0.4 ul<br>Accustart II MasterMix (2.5x) 8 uL<br>Template DNA 1ul (~10ng)	(2)
					94°C	45 sec	30x		
					50°C	60 sec			
					72°C	90 sec			
					72°C	10 min	1x		
					4°C	∞	hold		
rimM (Gilliamella single copy gene) PCR Round 1	ill_AMRimM_F	(5‘-TCGTCGGCAGCGTCAGATGTGTATAAGAGACAGGGCATTCGTGGWTGGCTCAG-3‘)	ill_AMRimM_R	(5‘-GTCTCGTGGGCTCGGAGATGTGTATAAGAGACAGACGTTCTGTTGCTCCAAAYGC-3‘)	94°C	3 min	1x	PCR reactions were conducted in 20 µL reactions of: Mol bio grade water 10.2ul<br>Forward (10uM) 0.4 ul<br>Reverse (10uM) 0.4 ul<br>Accustart II MasterMix(2.5x) 8 uL<br>Template DNA 1ul (~10ng)	(3)
					94°C	45 sec	30x		
					50°C	60 sec			
					72°C	90 sec			
					72°C	10 min	1x		
					4°C	∞	hold		
Bifido Groel (Bifidobacterium single copy gene) PCR Round 1	ill_Bif-groEL-F	(5‘-TCGTCGGCAGCGTCAGATGTGTATAAGAGACAGTCCGATTACGAYCGYGAGAAGCT-3‘)	ill_Bif-groEL-R	(5‘-GTCTCGTGGGCTCGGAGATGTGTATAAGAGACAGCSGCYTCGGTSGTCAGGAACAG-3‘)	94°C	3 min	1x	PCR reactions were conducted in 20 µL reactions of: Mol bio grade water 10.2ul<br>Forward (10uM) 0.4 ul<br>Reverse (10uM) 0.4 ul<br>Accustart II MasterMix (2.5x) 8 uL<br>Template DNA 1ul (~10ng)	(4)
					94°C	45 sec	30x		
					50°C	60 sec			
					72°C	90 sec			
					72°C	10 min	1x		
					4°C	∞	hold		
Barcoding primers (Nextera Round 2)	Hyb_Fnn_i5	(5'-AATGATACGGCGACCACCGAGATCTACAC NNNNNNNN TCGTCGGCAGCGTC -3')	Hyb_Rnn_i7	(5'-CAAGCAGAAGACGGCATACGAGAT NNN NNNNN GTCTCGTGGGCTCGG-3')	94°C	3 min	1x	PCR reactions were conducted in 25 µL reactions of: Mol bio grade water 6 ul<br>Index Primer 1 (N7xx) (5uM) 2 ul<br>Index Primer 2 (S5xx) (5uM) 2 ul<br>Accustart II MasterMix (2.5x) 12.5 uL<br>Template DNA(cleaned from PCR1) 5 ul	Illumina Nextera, "NNNNNNNN" signifies 8 b.p. unique sample barcode
					94°C	20 sec	10x		
					55°C	15 sec			
					72°C	60 sec			
					72°C	10 min	1x		
					4°C	∞	hold		
16S quantitative PCR	27F	(5’-AGAGTTTGATCCTGGCTCAG-3’)	355R	(5’-CTGCTGCCTCCCGTAGGAGT-3’)	95°C	10 min	x1	PCR reactions were conducted in 10 µL reactions of 5 µl 2x BioRad SYBR Master mix, 300 nM concentrations of each primer and ~50ng DNA.	(26)
					95°C	15 sec	x5		
					65-60°C (-1°C/cycle)	15 sec			
					68°C	20 sec			
					95°C	15 sec	x35		
					60°C	15 sec			
					68°C	20 sec			

Table S3. Plate map showing locations of samples yielding no product.

Key= no amplification in PCR 1 | read number too low to pass cut off

Day 0 (Collection ID#3)

16S	CTL					TYL				
	3-1SE1	3-4SE1	3-5SE1	3-8SE1	3-30BW1	3-11SE1	3-12SE1	3-13BW1	3-14BW1	3-15SE1
	3-1SE2	3-4SE2	3-5SE2	3-8SE2	3-30BW2	3-11SE2	3-12SE2	3-13BW2	3-14BW2	3-15SE2
	3-1SE3	3-4SE10	3-5SE3	3-8SE3	3-30BW3	3-11SE10	3-12SE3	3-13BW10	3-14BW3	3-15SE3
	3-1SE4	3-4SE4	3-5SE4	3-8SE4	3-30BW4	3-11SE4	3-12SE4	3-13BW4	3-14BW4	3-15SE4
	3-1SE5	3-4SE11	3-5SE5	3-8SE5	3-30BW5	3-11SE5	3-12SE5	3-13BW5	3-14BW5	3-15SE5
	3-1SE9	3-4SE9	3-5SE9	3-8SE9	3-30BW9	3-11SE9	3-12SE9	3-13BW9	3-14BW9	3-15SE9
	3-1SE10	3-4SE7	3-5SE7	3-8SE7	3-30BW7	3-11SE7	3-12SE7	3-13BW7	3-14BW7	3-15SE7
	3-1SE8	3-4SE8	3-5SE8	3-8SE8	3-30BW8	3-11SE8	3-12SE8	3-13BW8	3-14BW8	3-15SE8

rimM (Gilliamella)	CTL					TYL				
	**3-1SE1**	**3-4SE1**	3-5SE1	3-8SE1	3-30BW1	3-11SE1	3-12SE1	3-13BW1	3-14BW1	3-15SE1
	3-1SE2	3-4SE2	**3-5SE2**	3-8SE2	**3-30BW2**	**3-11SE2**	3-12SE2	**3-13BW2**	3-14BW2	3-15SE2
	3-1SE3	3-4SE10	3-5SE3	3-8SE3	**3-30BW3**	**3-11SE10**	3-12SE3	**3-13BW10**	3-14BW3	3-15SE3
	3-1SE4	3-4SE4	3-5SE4	3-8SE4	3-30BW4	3-11SE4	3-12SE4	3-13BW4	3-14BW4	3-15SE4
	**3-1SE5**	**3-4SE11**	**3-5SE5**	3-8SE5	**3-30BW5**	**3-11SE5**	3-12SE5	3-13BW5	3-14BW5	3-15SE5
	**3-1SE9**	3-4SE9	3-5SE9	3-8SE9	3-30BW9	3-11SE9	3-12SE9	**3-13BW9**	3-14BW9	3-15SE9
	**3-1SE10**	3-4SE7	3-5SE7	3-8SE7	**3-30BW7**	3-11SE7	3-12SE7	3-13BW7	3-14BW7	3-15SE7
	3-1SE8	**3-4SE8**	3-5SE8	3-8SE8	3-30BW8	3-11SE8	3-12SE8	3-13BW8	3-14BW8	3-15SE8

minD (Snodgrassella)	CTL					TYL				
	**3-1SE1**	3-4SE1	3-5SE1	3-8SE1	3-30BW1	3-11SE1	3-12SE1	3-13BW1	**3-14BW1**	3-15SE1
	3-1SE2	3-4SE2	3-5SE2	3-8SE2	3-30BW2	3-11SE2	3-12SE2	3-13BW2	3-14BW2	3-15SE2
	3-1SE3	3-4SE10	3-5SE3	3-8SE3	3-30BW3	3-11SE10	**3-12SE3**	**3-13BW10**	3-14BW3	**3-15SE3**
	3-1SE4	3-4SE4	3-5SE4	3-8SE4	3-30BW4	**3-11SE4**	3-12SE4	3-13BW4	**3-14BW4**	3-15SE4
	3-1SE5	**3-4SE11**	3-5SE5	3-8SE5	**3-30BW5**	3-11SE5	3-12SE5	**3-13BW5**	3-14BW5	3-15SE5
	**3-1SE9**	3-4SE9	3-5SE9	**3-8SE9**	3-30BW9	3-11SE9	3-12SE9	3-13BW9	3-14BW9	3-15SE9
	3-1SE10	**3-4SE7**	3-5SE7	3-8SE7	3-30BW7	3-11SE7	3-12SE7	3-13BW7	3-14BW7	3-15SE7
	3-1SE8	3-4SE8	3-5SE8	3-8SE8	3-30BW8	3-11SE8	3-12SE8	3-13BW8	3-14BW8	3-15SE8

groel (Bifido)	CTL					TYL				
	3-1SE1	3-4SE1	3-5SE1	3-8SE1	3-30BW1	3-11SE1	3-12SE1	3-13BW1	3-14BW1	3-15SE1
	3-1SE2	3-4SE2	**3-5SE2**	3-8SE2	**3-30BW2**	**3-11SE2**	3-12SE2	**3-13BW2**	3-14BW2	3-15SE2
	3-1SE3	3-4SE10	3-5SE3	3-8SE3	**3-30BW3**	**3-11SE10**	**3-12SE3**	**3-13BW10**	3-14BW3	3-15SE3
	3-1SE4	3-4SE4	**3-5SE4**	3-8SE4	3-30BW4	3-11SE4	3-12SE4	3-13BW4	3-14BW4	3-15SE4
	3-1SE5	3-4SE11	**3-5SE5**	3-8SE5	3-30BW5	3-11SE5	3-12SE5	3-13BW5	3-14BW5	3-15SE5
	**3-1SE9**	3-4SE9	3-5SE9	3-8SE9	3-30BW9	3-11SE9	3-12SE9	**3-13BW9**	3-14BW9	3-15SE9
	3-1SE10	3-4SE7	3-5SE7	3-8SE7	**3-30BW7**	3-11SE7	3-12SE7	3-13BW7	3-14BW7	3-15SE7
	3-1SE8	3-4SE8	3-5SE8	3-8SE8	3-30BW8	3-11SE8	3-12SE8	**3-13BW8**	3-14BW8	3-15SE8

Day 7 (Collection ID#4)

	CTL					TYL				
16S	4-1SE1	4-4SE1	4-5SE1	4-8SE1	4-30BW1	4-11SE1	4-12SE1	4-13BW1	4-14BW1	4-15SE1
	4-1SE2	4-4SE2	4-5SE2	4-8SE2	4-30BW2	4-11SE2	4-12SE2	4-13BW2	4-14BW2	4-15SE2
	4-1SE3	4-4SE3	4-5SE3	4-8SE3	4-30BW3	4-11SE3	4-12SE3	4-13BW3	4-14BW3	4-15SE3
	4-1SE4	4-4SE4	4-5SE4	4-8SE4	4-30BW4	4-11SE4	4-12SE4	4-13BW4	4-14BW4	4-15SE4
	4-1SE5	4-4SE5	4-5SE5	4-8SE5	4-30BW5	4-11SE5	4-12SE5	4-13BW5	4-14BW5	4-15SE5
	4-1SE6	4-4SE6	4-5SE6	4-8SE6	4-30BW6	4-11SE6	4-12SE6	4-13BW6	4-14BW6	4-15SE6
	4-1SE7	4-4SE7	4-5SE7	4-8SE7	4-30BW7	4-11SE7	4-12SE7	4-13BW7	4-14BW7	4-15SE7
	4-1SE8	4-4SE8	4-5SE8	4-8SE8	4-30BW8	4-11SE8	4-12SE8	4-13BW8	4-14BW8	4-15SE8
rimM (Gilliamella)	4-1SE1	4-4SE1	4-5SE1	4-8SE1	4-30BW1	4-11SE1	4-12SE1	4-13BW1	4-14BW1	4-15SE1
	4-1SE2	4-4SE2	4-5SE2	4-8SE2	4-30BW2	4-11SE2	4-12SE2	4-13BW2	4-14BW2	4-15SE2
	4-1SE3	4-4SE3	4-5SE3	4-8SE3	4-30BW3	4-11SE3	4-12SE3	4-13BW3	4-14BW3	4-15SE3
	4-1SE4	4-4SE4	4-5SE4	4-8SE4	4-30BW4	4-11SE4	4-12SE4	4-13BW4	4-14BW4	4-15SE4
	4-1SE5	4-4SE5	4-5SE5	4-8SE5	4-30BW5	4-11SE5	4-12SE5	4-13BW5	4-14BW5	4-15SE5
	4-1SE6	4-4SE6	4-5SE6	4-8SE6	4-30BW6	4-11SE6	4-12SE6	4-13BW6	4-14BW6	4-15SE6
	4-1SE7	4-4SE7	4-5SE7	4-8SE7	4-30BW7	4-11SE7	4-12SE7	4-13BW7	4-14BW7	4-15SE7
	4-1SE8	4-4SE8	4-5SE8	4-8SE8	4-30BW8	4-11SE8	4-12SE8	4-13BW8	4-14BW8	4-15SE8
minD (Snodgrassella)	4-1SE1	4-4SE1	4-5SE1	4-8SE1	4-30BW1	4-11SE1	4-12SE1	4-13BW1	4-14BW1	4-15SE1
	4-1SE2	4-4SE2	4-5SE2	4-8SE2	4-30BW2	4-11SE2	4-12SE2	4-13BW2	4-14BW2	4-15SE2
	4-1SE3	4-4SE3	4-5SE3	4-8SE3	4-30BW3	4-11SE3	4-12SE3	4-13BW3	4-14BW3	4-15SE3
	4-1SE4	4-4SE4	4-5SE4	4-8SE4	4-30BW4	4-11SE4	4-12SE4	4-13BW4	4-14BW4	4-15SE4
	4-1SE5	4-4SE5	4-5SE5	4-8SE5	4-30BW5	4-11SE5	4-12SE5	4-13BW5	4-14BW5	4-15SE5
	4-1SE6	4-4SE6	4-5SE6	4-8SE6	4-30BW6	4-11SE6	4-12SE6	4-13BW6	4-14BW6	4-15SE6
	4-1SE7	4-4SE7	4-5SE7	4-8SE7	4-30BW7	4-11SE7	4-12SE7	4-13BW7	4-14BW7	4-15SE7
	4-1SE8	4-4SE8	4-5SE8	4-8SE8	4-30BW8	4-11SE8	4-12SE8	4-13BW8	4-14BW8	4-15SE8
groel (Bifido)	4-1SE1	4-4SE1	4-5SE1	4-8SE1	4-30BW1	4-11SE1	4-12SE1	4-13BW1	4-14BW1	4-15SE1
	4-1SE2	4-4SE2	4-5SE2	4-8SE2	4-30BW2	4-11SE2	4-12SE2	4-13BW2	4-14BW2	4-15SE2
	4-1SE3	4-4SE3	4-5SE3	4-8SE3	4-30BW3	4-11SE3	4-12SE3	4-13BW3	4-14BW3	4-15SE3
	4-1SE4	4-4SE4	4-5SE4	4-8SE4	4-30BW4	4-11SE4	4-12SE4	4-13BW4	4-14BW4	4-15SE4
	4-1SE5	4-4SE5	4-5SE5	4-8SE5	4-30BW5	4-11SE5	4-12SE5	4-13BW5	4-14BW5	4-15SE5
	4-1SE6	4-4SE6	4-5SE6	4-8SE6	4-30BW6	4-11SE6	4-12SE6	4-13BW6	4-14BW6	4-15SE6
	4-1SE7	4-4SE7	4-5SE7	4-8SE7	4-30BW7	4-11SE7	4-12SE7	4-13BW7	4-14BW7	4-15SE7
	4-1SE8	4-4SE8	4-5SE8	4-8SE8	4-30BW8	4-11SE8	4-12SE8	4-13BW8	4-14BW8	4-15SE8

Day 14 (Collection ID#5)

16S

	CTL						TYL					
	5-1SE1	5-4SE1	5-5SE1	5-8SE1	5-30BW1		5-11SE1	5-12SE1	5-13BW1	5-14BW1	5-15SE1	
	5-1SE2	5-4SE2	5-5SE2	5-8SE2	5-30BW2		5-11SE2	5-12SE2	5-13BW2	5-14BW2	5-15SE2	
	5-1SE3	5-4SE3	5-5SE3	5-8SE3	5-30BW3		5-11SE3	5-12SE3	5-13BW3	5-14BW3	5-15SE3	
	5-1SE4	5-4SE4	5-5SE4	5-8SE4	5-30BW4		5-11SE4	5-12SE4	5-13BW4	5-14BW4	5-15SE4	
	5-1SE5	5-4SE5	5-5SE5	5-8SE5	5-30BW5		5-11SE5	5-12SE5	5-13BW5	5-14BW5	5-15SE5	
	5-1SE6	5-4SE6	5-5SE6	5-8SE6	5-30BW6		5-11SE6	5-12SE6	5-13BW6	5-14BW6	5-15SE6	
	5-1SE7	5-4SE7	5-5SE7	5-8SE7	5-30BW7		5-11SE7	5-12SE7	5-13BW7	5-14BW7	5-15SE7	
	5-1SE8	5-4SE8	5-5SE8	5-8SE8	5-30BW8		5-11SE8	5-12SE8	5-13BW8	5-14BW8	5-15SE8	

rimM (Gilliamella)

minD (Snodgrassella)

groel (Bifido)

Day 21 (Collection ID#6)

	CTL					TYL				
16S	6-1SE1	6-4SE1	6-5SE1	6-8SE1	6-30BW1	6-11SE1	6-12SE1	6-13BW1	6-14BW1	6-15SE1
	6-1SE2	6-4SE2	6-5SE2	6-8SE2	6-30BW2	6-11SE2	6-12SE2	6-13BW2	6-14BW2	6-15SE2
	6-1SE3	6-4SE3	6-5SE3	6-8SE3	6-30BW3	6-11SE3	6-12SE3	6-13BW3	6-14BW3	6-15SE3
	6-1SE4	6-4SE4	6-5SE4	6-8SE4	6-30BW4	6-11SE4	6-12SE4	6-13BW4	6-14BW4	6-15SE4
	6-1SE5	6-4SE5	6-5SE5	6-8SE5	6-30BW5	6-11SE5	6-12SE5	6-13BW5	6-14BW5	6-15SE5
	6-1SE9	6-4SE9	6-5SE9	6-8SE9	6-30BW9	6-11SE9	6-12SE9	6-13BW9	6-14BW9	6-15SE9
	6-1SE7	6-4SE7	6-5SE7	6-8SE7	6-30BW7	6-11SE7	6-12SE7	6-13BW7	6-14BW7	6-15SE7
	6-1SE8	6-4SE8	6-5SE8	6-8SE8	6-30BW8	6-11SE8	6-12SE8	6-13BW8	6-14BW8	6-15SE8
rimM (Gilliamella)	6-1SE1	6-4SE1	6-5SE1	6-8SE1	6-30BW1	6-11SE1	6-12SE1	6-13BW1	6-14BW1	6-15SE1
	6-1SE2	6-4SE2	6-5SE2	6-8SE2	6-30BW2	6-11SE2	6-12SE2	6-13BW2	6-14BW2	6-15SE2
	6-1SE3	6-4SE3	6-5SE3	6-8SE3	6-30BW3	6-11SE3	6-12SE3	6-13BW3	6-14BW3	6-15SE3
	6-1SE4	6-4SE4	6-5SE4	6-8SE4	6-30BW4	6-11SE4	6-12SE4	6-13BW4	6-14BW4	6-15SE4
	6-1SE5	6-4SE5	6-5SE5	6-8SE5	6-30BW5	6-11SE5	6-12SE5	6-13BW5	6-14BW5	6-15SE5
	6-1SE9	6-4SE9	6-5SE9	6-8SE9	6-30BW9	6-11SE9	6-12SE9	6-13BW9	6-14BW9	6-15SE9
	6-1SE7	6-4SE7	6-5SE7	6-8SE7	6-30BW7	6-11SE7	6-12SE7	6-13BW7	6-14BW7	6-15SE7
	6-1SE8	6-4SE8	6-5SE8	6-8SE8	6-30BW8	6-11SE8	6-12SE8	6-13BW8	6-14BW8	6-15SE8
minD (Snodgrassella)	6-1SE1	6-4SE1	6-5SE1	6-8SE1	6-30BW1	6-11SE1	6-12SE1	6-13BW1	6-14BW1	6-15SE1
	6-1SE2	6-4SE2	6-5SE2	6-8SE2	6-30BW2	6-11SE2	6-12SE2	6-13BW2	6-14BW2	6-15SE2
	6-1SE3	6-4SE3	6-5SE3	6-8SE3	6-30BW3	6-11SE3	6-12SE3	6-13BW3	6-14BW3	6-15SE3
	6-1SE4	6-4SE4	6-5SE4	6-8SE4	6-30BW4	6-11SE4	6-12SE4	6-13BW4	6-14BW4	6-15SE4
	6-1SE5	6-4SE5	6-5SE5	6-8SE5	6-30BW5	6-11SE5	6-12SE5	6-13BW5	6-14BW5	6-15SE5
	6-1SE9	6-4SE9	6-5SE9	6-8SE9	6-30BW9	6-11SE9	6-12SE9	6-13BW9	6-14BW9	6-15SE9
	6-1SE7	6-4SE7	6-5SE7	6-8SE7	6-30BW7	6-11SE7	6-12SE7	6-13BW7	6-14BW7	6-15SE7
	6-1SE8	6-4SE8	6-5SE8	6-8SE8	6-30BW8	6-11SE8	6-12SE8	6-13BW8	6-14BW8	6-15SE8
groel (Bifido)	6-1SE1	6-4SE1	6-5SE1	6-8SE1	6-30BW1	6-11SE1	6-12SE1	6-13BW1	6-14BW1	6-15SE1
	6-1SE2	6-4SE2	6-5SE2	6-8SE2	6-30BW2	6-11SE2	6-12SE2	6-13BW2	6-14BW2	6-15SE2
	6-1SE3	6-4SE3	6-5SE3	6-8SE3	6-30BW3	6-11SE3	6-12SE3	6-13BW3	6-14BW3	6-15SE3
	6-1SE4	6-4SE4	6-5SE4	6-8SE4	6-30BW4	6-11SE4	6-12SE4	6-13BW4	6-14BW4	6-15SE4
	6-1SE5	6-4SE5	6-5SE5	6-8SE5	6-30BW5	6-11SE5	6-12SE5	6-13BW5	6-14BW5	6-15SE5
	6-1SE9	6-4SE9	6-5SE9	6-8SE9	6-30BW9	6-11SE9	6-12SE9	6-13BW9	6-14BW9	6-15SE9
	6-1SE7	6-4SE7	6-5SE7	6-8SE7	6-30BW7	6-11SE7	6-12SE7	6-13BW7	6-14BW7	6-15SE7
	6-1SE8	6-4SE8	6-5SE8	6-8SE8	6-30BW8	6-11SE8	6-12SE8	6-13BW8	6-14BW8	6-15SE8

Day 49 (Collection ID#8)

	CTL					TYL				
16S	8-1SE1	8-4SE1	8-5SE1	8-8SE1	8-30BW1	8-11SE1	8-12SE1	8-13BW1	8-14BW1	8-15SE1
	8-1SE2	8-4SE2	8-5SE2	8-8SE2	8-30BW2	8-11SE2	8-12SE2	8-13BW2	8-14BW2	8-15SE2
	8-1SE3	8-4SE3	8-5SE3	8-8SE3	8-30BW3	8-11SE3	8-12SE3	8-13BW3	8-14BW3	8-15SE3
	8-1SE4	8-4SE4	8-5SE4	8-8SE4	8-30BW4	8-11SE4	8-12SE4	8-13BW4	8-14BW4	8-15SE4
	8-1SE5	8-4SE5	8-5SE5	8-8SE5	8-30BW5	8-11SE5	8-12SE5	8-13BW5	8-14BW5	8-15SE5
	8-1SE6	8-4SE6	8-5SE6	8-8SE6	8-30BW6	8-11SE6	8-12SE6	8-13BW6	8-14BW6	8-15SE6
	8-1SE7	8-4SE7	8-5SE7	8-8SE7	8-30BW7	8-11SE7	8-12SE7	8-13BW7	8-14BW7	8-15SE7
	8-1SE8	8-4SE8	8-5SE8	8-8SE8	8-30BW8	8-11SE8	8-12SE8	8-13BW8	8-14BW8	8-15SE8

rimM (Gilliamella)	8-1SE1	8-4SE1	8-5SE1	8-8SE1	8-30BW1	8-11SE1	8-12SE1	8-13BW1	8-14BW1	8-15SE1
	8-1SE2	8-4SE2	8-5SE2	8-8SE2	8-30BW2	8-11SE2	8-12SE2	8-13BW2	8-14BW2	8-15SE2
	8-1SE3	8-4SE3	8-5SE3	8-8SE3	8-30BW3	8-11SE3	8-12SE3	8-13BW3	8-14BW3	8-15SE3
	8-1SE4	8-4SE4	8-5SE4	8-8SE4	8-30BW4	8-11SE4	8-12SE4	8-13BW4	8-14BW4	8-15SE4
	8-1SE5	8-4SE5	8-5SE5	8-8SE5	8-30BW5	8-11SE5	8-12SE5	8-13BW5	8-14BW5	8-15SE5
	8-1SE6	8-4SE6	8-5SE6	8-8SE6	8-30BW6	8-11SE6	8-12SE6	8-13BW6	8-14BW6	8-15SE6
	8-1SE7	8-4SE7	8-5SE7	8-8SE7	8-30BW7	8-11SE7	8-12SE7	8-13BW7	8-14BW7	8-15SE7
	8-1SE8	8-4SE8	8-5SE8	8-8SE8	8-30BW8	8-11SE8	8-12SE8	8-13BW8	8-14BW8	8-15SE8

minD (Snodgrassella)	8-1SE1	8-4SE1	8-5SE1	8-8SE1	8-30BW1	8-11SE1	8-12SE1	8-13BW1	8-14BW1	8-15SE1
	8-1SE2	8-4SE2	8-5SE2	8-8SE2	8-30BW2	8-11SE2	8-12SE2	8-13BW2	8-14BW2	8-15SE2
	8-1SE3	8-4SE3	8-5SE3	8-8SE3	8-30BW3	8-11SE3	8-12SE3	8-13BW3	8-14BW3	8-15SE3
	8-1SE4	8-4SE4	8-5SE4	8-8SE4	8-30BW4	8-11SE4	8-12SE4	8-13BW4	8-14BW4	8-15SE4
	8-1SE5	8-4SE5	8-5SE5	8-8SE5	8-30BW5	8-11SE5	8-12SE5	8-13BW5	8-14BW5	8-15SE5
	8-1SE6	8-4SE6	8-5SE6	8-8SE6	8-30BW6	8-11SE6	8-12SE6	8-13BW6	8-14BW6	8-15SE6
	8-1SE7	8-4SE7	8-5SE7	8-8SE7	8-30BW7	8-11SE7	8-12SE7	8-13BW7	8-14BW7	8-15SE7
	8-1SE8	8-4SE8	8-5SE8	8-8SE8	8-30BW8	8-11SE8	8-12SE8	8-13BW8	8-14BW8	8-15SE8

groel (Bifido)	8-1SE1	8-4SE1	8-5SE1	8-8SE1	8-30BW1	8-11SE1	8-12SE1	8-13BW1	8-14BW1	8-15SE1
	8-1SE2	8-4SE2	8-5SE2	8-8SE2	8-30BW2	8-11SE2	8-12SE2	8-13BW2	8-14BW2	8-15SE2
	8-1SE3	8-4SE3	8-5SE3	8-8SE3	8-30BW3	8-11SE3	8-12SE3	8-13BW3	8-14BW3	8-15SE3
	8-1SE4	8-4SE4	8-5SE4	8-8SE4	8-30BW4	8-11SE4	8-12SE4	8-13BW4	8-14BW4	8-15SE4
	8-1SE5	8-4SE5	8-5SE5	8-8SE5	8-30BW5	8-11SE5	8-12SE5	8-13BW5	8-14BW5	8-15SE5
	8-1SE6	8-4SE6	8-5SE6	8-8SE6	8-30BW6	8-11SE6	8-12SE6	8-13BW6	8-14BW6	8-15SE6
	8-1SE7	8-4SE7	8-5SE7	8-8SE7	8-30BW7	8-11SE7	8-12SE7	8-13BW7	8-14BW7	8-15SE7
	8-1SE8	8-4SE8	8-5SE8	8-8SE8	8-30BW8	8-11SE8	8-12SE8	8-13BW8	8-14BW8	8-15SE8

Table S4: Accession numbers of sequences used for single copy gene alignments

minD (Snodgrassella)
>CP007446.1:381779-382292 Snodgrassella alvi wkB2, complete genome
>MEIX01000013.1:38471-38984 Snodgrassella alvi strain MS1-3 NODE_11_length_40960_cov_26.580835, whole genome shotgun sequence
>MEIN01000012.1:43939-44452 Snodgrassella alvi strain wkB9 1196083.66_ctg7180000000394, whole genome shotgun sequence
>NAGX01000079.1:2389-2902 Snodgrassella alvi strain A12 NODE_136, whole genome shotgun sequence
>NAHJ01000039.1:2120-2633 Snodgrassella alvi strain N-W7 NODE_24, whole genome shotgun sequence
>QGLS01000007.1:39467-39980 Snodgrassella alvi strain ESL0196 Ga0133560_107, whole genome shotgun sequence
>NXEN01000142.1:2031-2544 Snodgrassella alvi strain E1 Contig_203, whole genome shotgun sequence
>NAHF01000104.1:2115-2628 Snodgrassella alvi strain A5 NODE_17, whole genome shotgun sequence
>NAHE01000079.1:27066-27579 Snodgrassella alvi strain A3 NODE_31, whole genome shotgun sequence
>NAHC01000059.1:28076-28589 Snodgrassella alvi strain A2 NODE_26, whole genome shotgun sequence
>NAGZ01000105.1:2115-2628 Snodgrassella alvi strain A11 NODE_18, whole genome shotgun sequence
>NAHK01000098.1:27159-27671 Snodgrassella alvi strain N9 NODE_32, whole genome shotgun sequence
>NAHH01000033.1:2198-2711 Snodgrassella alvi strain A-9-24 NODE_30, whole genome shotgun sequence
>NAHG01000065.1:2198-2711 Snodgrassella alvi strain A-5-24 NODE_108, whole genome shotgun sequence
>NAHD01000045.1:2198-2711 Snodgrassella alvi strain A-2-12 NODE_31, whole genome shotgun sequence
>NAHB01000036.1:38789-39302 Snodgrassella alvi strain A-1-12 NODE_23, whole genome shotgun sequence
>NAHA01000028.1:2198-2711 Snodgrassella alvi strain A-11-12 NODE_63, whole genome shotgun sequence
>NAGY01000040.1:38789-39302 Snodgrassella alvi strain A-10-12 NODE_24, whole genome shotgun sequence
>AVQL01000444.1:7591-8104 Snodgrassella alvi SCGC AB-598-J21 SCG598J21_12826, whole genome shotgun sequence
>NAHN01000056.1:38530-39042 Snodgrassella alvi strain N-S3 NODE_24, whole genome shotgun sequence
>MEIJ01000005.1:38956-39469 Snodgrassella alvi strain wkB332 1196083.70_ctg7180000000224, whole genome shotgun sequence
>MEII01000012.1:39218-39731 Snodgrassella alvi strain wkB339 1196083.71_ctg7180000000833, whole genome shotgun sequence
>NAGW01000059.1:2131-2644 Snodgrassella alvi strain Aw-18 NODE_30, whole genome shotgun sequence
>MVDP01000043.1:2178-2691 Snodgrassella alvi strain Aw-20 NODE_23, whole genome shotgun sequence
>NAHQ01000113.1:40085-40598 Snodgrassella alvi strain N-23 NODE_16, whole genome shotgun sequence
>NAHP01000046.1:2342-2855 Snodgrassella alvi strain N-S5 NODE_32, whole genome shotgun sequence
>NAHO01000015.1:2343-2856 Snodgrassella alvi strain N-S4 NODE_24, whole genome shotgun sequence
>NAHM01000045.1:27166-27679 Snodgrassella alvi strain N-S2 NODE_29, whole genome shotgun sequence
>NAHL01000092.1:72259-72772 Snodgrassella alvi strain N-S1 NODE_7, whole genome shotgun sequence
>NAHI01000053.1:40085-40598 Snodgrassella alvi strain N-W4 NODE_23, whole genome shotgun sequence
>MEIW01000110.1:2086-2599 Snodgrassella alvi strain PEB0178 NODE_63_length_30946_cov_31.719900, whole genome shotgun sequence
>MEIV01000036.1:2062-2575 Snodgrassella alvi strain PEB0171 NODE_25_length_30757_cov_38.671654, whole genome shotgun sequence
>MEIO01000003.1:38357-38870 Snodgrassella alvi strain WF3-3 1196083.65_ctg7180000001694, whole genome shotgun sequence
>MDVG01000047.1:45343-45856 Snodgrassella alvi strain Fer4-2 1196083.54_ctg2833, whole genome shotgun sequence
>MEIU01000042.1:2811-3324 Snodgrassella alvi strain HK3 1196083.58_ctg7180000000279, whole genome shotgun sequence
>MEIT01000099.1:3258-3771 Snodgrassella alvi strain HK9x 1196083.59_ctg7180000000512, whole genome shotgun sequence
>MDUZ01000057.1:2156-2669 Snodgrassella alvi strain Pens2-2-5 1196083.22_NODE_22, whole genome shotgun sequence
>MDVB01000003.1:2430-2943 Snodgrassella alvi strain App2-2 1196083.48_ctg0892, whole genome shotgun sequence
>MEIS01000057.1:2805-3318 Snodgrassella alvi strain Nev3CBA3 1196083.61_ctg4603, whole genome shotgun sequence
>MEIR01000069.1:2197-2710 Snodgrassella alvi strain Nev4-2 1196083.62_ctg7180000001591, whole genome shotgun sequence
>MDVJ01000046.1:96433-96946 Snodgrassella alvi strain Gris3-4 1196083.57_ctg7180000001169, whole genome shotgun sequence
>MDVI01000032.1:2451-2964 Snodgrassella alvi strain Gris1-6 1196083.56_ctg7180000000365, whole genome shotgun sequence
>MDVH01000054.1:3708-4221 Snodgrassella alvi strain Gris1-3 1196083.55_ctg7180000000218, whole genome shotgun sequence
>MDVD01000058.1:39906-40419 Snodgrassella alvi strain App6-4 1196083.50_ctg1098, whole genome shotgun sequence
>MDVC01000089.1:7701-8214 Snodgrassella alvi strain App4-8 1196083.49_ctg0880, whole genome shotgun sequence
>MDUY01000017.1:2171-2684 Snodgrassella alvi strain Gris2-3-4 1196083.20_NODE_17, whole genome shotgun sequence
>JFZW01000005.1:7783-8296 Snodgrassella alvi strain wkB12 wkB12_scaf005, whole genome shotgun sequence
>JFZV01000014.1:7742-8255 Snodgrassella alvi strain wkB29 wkB29_scaf014, whole genome shotgun sequence
>MEIQ01000046.1:38726-39239 Snodgrassella alvi strain Occ4-2 1196083.63_ctg7180000000456, whole genome shotgun sequence
>MEIP01000016.1:37355-37868 Snodgrassella alvi strain Ruf1-X 1196083.64_ctg7180000000750, whole genome shotgun sequence
>MDVF01000024.1:2895-3408 Snodgrassella alvi strain Fer2-2 1196083.53_ctg7180000002672, whole genome shotgun sequence
>MDVE01000065.1:3027-3540 Snodgrassella alvi strain Fer1-2 1196083.52_ctg7180000001848, whole genome shotgun sequence
>MDVA01000012.1:34866-35379 Snodgrassella alvi strain Snod2-1-5 1196083.23_NODE_15, whole genome shotgun sequence
>MEIM01000007.1:37597-38110 Snodgrassella alvi strain wkB237 1196083.67_ctg7180000000080, whole genome shotgun sequence
>MEIL01000017.1:37554-38067 Snodgrassella alvi strain wkB273 1196083.68_ctg7180000000522, whole genome shotgun sequence
>MEIK01000001.1:2198-2711 Snodgrassella alvi strain wkB298 1196083.69_ctg7180000001191, whole genome shotgun sequence
>JAIL01000242.1:1945-2457 Snodgrassella alvi SCGC AB-598-O02 SCG598O02_12008, whole genome shotgun sequence
>MN385157.1 Snodgrassella alvi strain C14 septum sit-determining protein MinD (minD) gene, partial cds
>MN385153.1 Snodgrassella alvi strain I2 septum sit-determining protein MinD (minD) gene, partial cds
>MN385153.1 Snodgrassella alvi strain I2 septum sit-determining protein MinD (minD) gene, partial cds
>MN385154.1 Snodgrassella alvi strain I49 septum sit-determining protein MinD (minD) gene, partial cds
>MN385156.1 Snodgrassella alvi strain C11 septum sit-determining protein MinD (minD) gene, partial cds

rimM (Gilliamella)
>JAIM01000005.1:46279-46579 Gilliamella apicola SCGC AB-598-B02 SCG598B02_13034, whole genome shotgun sequence
>VMHM01000002.1:35861-36161 Gilliamella apicola strain W8127 NODE_2_length_246611_cov_246.806365, whole genome shotgun sequence
>NARZ01000070.1:38345-38645 Gilliamella apicola strain N6 NODE_13, whole genome shotgun sequence

>LZGJ01000024.1:220893-221193 Gilliamella apicola strain P54G Gilliamella_apicola_P54G_contig31, whole genome shotgun sequence
>QICT01000004.1:38340-38640 Gilliamella apicola strain DSM 104097 Ga0215725_104, whole genome shotgun sequence
>LZGN01000043.1:64573-64873 Gilliamella apicola strain wkB308 Gilliamella_apicola_wkB308_contig6, whole genome shotgun sequence
>LZHP01000020.1:52294-52594 Gilliamella apicola strain Nev6-6 Gilliamella_apicola_Nev6-6_contig23, whole genome shotgun sequence
>LZGO01000045.1:31864-32162 Gilliamella apicola strain wkB292 Gilliamella_apicola_wkB292_contig5, whole genome shotgun sequence
>WTEV01000002.1:189784-190084 Gilliamella sp. Pas-s25 NODE_2_length_191684_cov_392.390315, whole genome shotgun sequence
>LZGX01000012.1:19326-19626 Gilliamella apicola strain Fer1-1 Gilliamella_apicola_Fer1-1_contig109, whole genome shotgun sequence
>NASL01000010.1:28944-29242 Gilliamella apis strain AM1 NODE_10, whole genome shotgun sequence
>LZHO01000088.1:43780-44080 Gilliamella apicola strain Nev5-1 Gilliamella_apicola_Nev5-1_contig89, whole genome shotgun sequence
>LZGZ01000018.1:8214-8514 Gilliamella apicola strain Fer4-1 Gilliamella_apicola_Fer4-1_contig114, whole genome shotgun sequence
>QGLO01000007.1:171119-171417 Gilliamella apis strain ESL0172 Ga0133556_108, whole genome shotgun sequence
>LZEI01000021.1:125896-126196 Gilliamella apicola strain wkB72 Gilliamella_apicola_wkB72_contig28, whole genome shotgun sequence
>NASV01000005.1:34267-34565 Gilliamella apis strain N-G4 NODE_5, whole genome shotgun sequence
>LZHK01000103.1:11560-11860 Gilliamella apicola strain Imp1-1 Gilliamella_apicola_Imp1-1_contig64, whole genome shotgun sequence
>JAIO01000001.1:26512-26810 Gilliamella apis SCGC AB-598-P17 SCG598P17_12889, whole genome shotgun sequence
>LZGT01000019.1:68890-69190 Gilliamella apicola strain App6-5 Gilliamella_apicola_App6-5_contig2, whole genome shotgun sequence
>LZHQ01000034.1:136583-136883 Gilliamella apicola strain Occ3-1 Gilliamella_apicola_Occ3-1_contig7, whole genome shotgun sequence
>LZHI01000068.1:46646-46946 Gilliamella apicola strain Choc6-1 Gilliamella_apicola_Choc6-1_contig9, whole genome shotgun sequence
>LZHA01000079.1:57633-57933 Gilliamella apicola strain Gris1-4 Gilliamella_apicola_Gris1-4_contig80, whole genome shotgun sequence

groEL (Bifido)
>CP007287: Bifidobacterium coryneforme strain LMG18911
>KC776067: Bifidobacterium asteroides strain Hma3
>CP006018: Bifidobacterium indicum LMG 11587 = DSM 20214
>CP017696: Bifidobacterium asteroides strain DSM 20089
>NZ_AWUN01000004.1: Bifidobacterium sp. 7101
>NZ_KQ033859.1: Bifidobacterium asteroides strain Bin2
>NZ_KQ033885.1:Bifidobacterium asteroides strain Bin7
>NZ_AWUO01000001.1: Bifidobacterium sp. A11
>NZ_KQ034040.1: Bifidobacterium sp. A11
>NPOQ01000009.1: Bifidobacterium sp. strain WKB344
>JXBX01000009.1:668167-668586 Bifidobacterium coryneforme strain Bma6 contig009, whole genome shotgun sequence
>QGLI01000005.1:811036-811455 Bifidobacterium indicum strain ESL0197 Ga0133550_15, whole genome shotgun sequence
>PCHJ01000015.1:349457-349876 Bifidobacterium asteroides strain 1460B Contig_15, whole genome shotgun sequence
>VMHK01000004.1:53340-53759 Bifidobacterium asteroides strain W8102 NODE_4_length_169663_cov_292.432179, whole genome shotgun sequence
>QGLK01000004.1:345193-345612 Bifidobacterium asteroides strain ESL0199 Ga0133552_14, whole genome shotgun sequence
>WKKW01000001.1:228359-228777 Bifidobacterium asteroides strain VRA_9sq_n contig01, whole genome shotgun sequence
>QGLH01000003.1:441854-442273 Bifidobacterium asteroides strain ESL0170 Ga0133549_13, whole genome shotgun sequence
>QGLL01000008.1:64003-64422 Bifidobacterium asteroides strain ESL0200 Ga0133553_109, whole genome shotgun sequence
>QGLJ01000007.1:920920-921339 Bifidobacterium asteroides strain ESL0198 Ga0133551_107, whole genome shotgun sequence
>VMHJ01000001.1:924171-924590 Bifidobacterium asteroides strain W8111 NODE_1_length_1144558_cov_270.985906, whole genome shotgun sequence
>NPOR01000049.1:22702-23121 Bifidobacterium sp. wkB338 ctg7180000000346, whole genome shotgun sequence
